# Cost-effectiveness of BPaL-based and 9-month modified all-oral short treatment regimens for rifampicin-resistant tuberculosis in Belarus

**DOI:** 10.1371/journal.pgph.0005872

**Published:** 2026-07-23

**Authors:** Natalia Yatskevich

**Affiliations:** Republican Scientific and Practical Center for Pulmonology and Tuberculosis, Minsk, Belarus; Faculty of Medicine, Khon Kaen University, THAILAND

## Abstract

Belarus remains a high-burden country for multidrug-/rifampicin-resistant tuberculosis (MDR/RR-TB), including pre-extensively drug-resistant TB (pre-XDR-TB). BPaL-based and modified 9-month short treatment regimens (mSTR), implemented under operational research conditions, have demonstrated improved clinical outcomes, but evidence on their cost-effectiveness in real-world settings remains limited. We evaluated the cost-effectiveness of BPaL-based regimens containing bedaquiline, pretomanid, and linezolid with either moxifloxacin or clofazimine (BPaL(M/C)); mSTR composed of bedaquiline, levofloxacin, linezolid, clofazimine, cycloserine, or delamanid; and standard of care (SOC; ≥ 18-month regimens) for MDR/RR-TB and pre-XDR-TB in Belarus over a 20-year horizon using a cohort-based Markov model. Clinical effectiveness, safety, and cost inputs were derived from operational research and WHO data. Health outcomes were measured in quality-adjusted life-years (QALYs), costs were expressed in 2022 USD, and both were discounted at 3% annually. Incremental costs, QALYs, and incremental net monetary benefits (INMB) were estimated. Univariate, probabilistic, and conservative scenario analyses were conducted. A scale-up analysis assessed national budgetary implications. Probabilistic sensitivity analysis showed that both shorter regimens were cost-effective compared with SOC at the Belarus-specific willingness-to-pay threshold, with mean [IQR] INMB of USD 27,586 [16,248; 37,645] for BPaL(M/C) and USD 23,488 [10,887; 35,092] for mSTR versus SOC. In the base case, BPaL(M/C) was the most cost-effective strategy, with lower mean costs (USD 8,155) and higher effectiveness (10.82 QALYs) than SOC (USD 20,757; 8.91 QALYs). mSTR was also cost-effective compared with SOC but less effective than BPaL(M/C). Results were robust across sensitivity and scenario analyses. Under conservative assumptions of identical clinical outcomes, shorter regimens remained cost-saving. The scale-up analysis showed that expanding use of shorter regimens in Belarus could generate annual TB programme budget savings exceeding USD 9.8 million. BPaL(M/C) and mSTR are cost-effective alternatives to SOC in Belarus, offering substantial economic benefits at both patient and programmatic levels.

## Introduction

Multidrug-resistant/rifampicin-resistant tuberculosis (MDR/RR-TB), including pre-extensively drug-resistant tuberculosis (pre-XDR-TB), remains a major global health challenge, particularly in Eastern Europe. Belarus is among the countries with a high burden of MDR/RR-TB and pre-XDR-TB [1].

Recent advances in the treatment of drug-resistant TB include the introduction of shorter, all-oral regimens. These include BPaL-based regimens (bedaquiline, pretomanid, and linezolid), as well as other shorter treatment regimens such as modified 9-month shorter treatment regimens (mSTR) composed of bedaquiline, levofloxacin, linezolid, clofazimine, cycloserine, or delamanid, which have been implemented under operational research conditions [2,3].

Clinical trials and programmatic experience suggest that these regimens are associated with improved treatment outcomes and reduced treatment duration compared with conventional long regimens. Evidence from trial-based and modelling studies indicates that shorter regimens may reduce treatment-related costs; however, their cost-effectiveness under real-world programmatic conditions remains insufficiently established, particularly in Eastern Europe [4].

The objective of this study was to evaluate the cost-effectiveness of BPaL-based regimens and a modified 9-month all-oral short treatment regimens (mSTR), compared with standard of care (SOC; ≥ 18-month regimens) and with each other, by simulating the clinical course and economic consequences of treating patients with MDR/RR-TB and pre-XDR-TB in Belarus using operational research and WHO data.

## Methods

**Fig 1:**
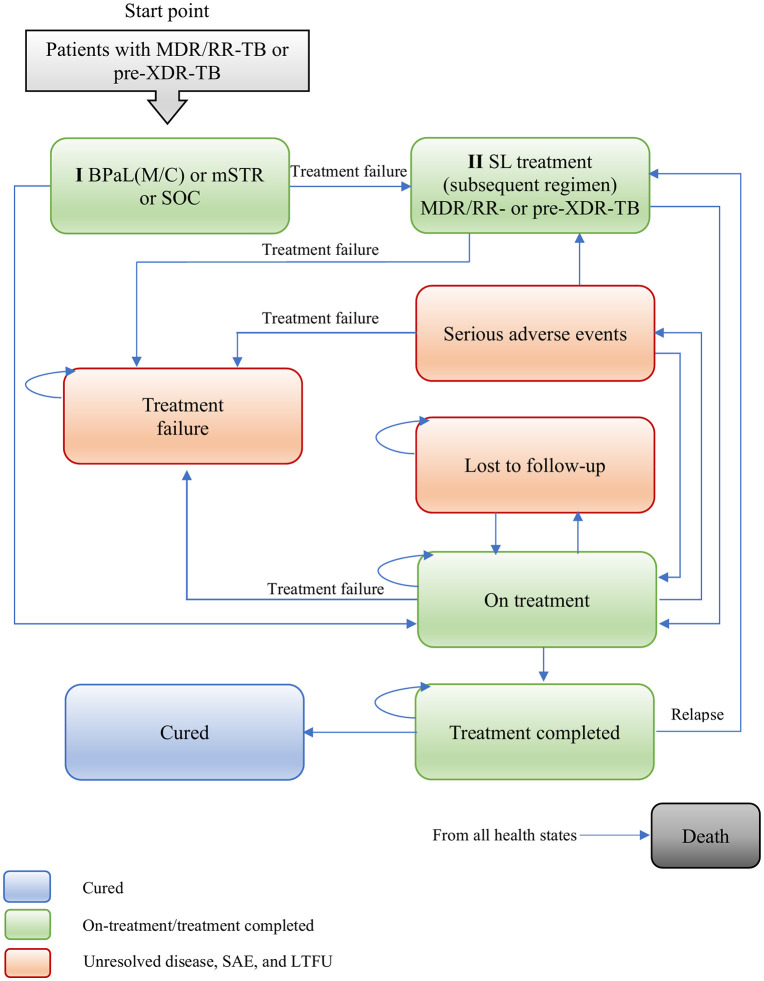
Markov model diagram. The initial strategy state (“BPaL(M/C), mSTR, or SOC”) denotes the treatment regimen assigned at model entry and determines regimen-specific duration, costs, effectiveness, and safety parameters. The “On treatment” state represents patients actively receiving therapy within the assigned regimen or subsequent second-line treatment. Arrows from “Serious adverse events” and “Lost to follow-up” to “On treatment” indicate continuation or resumption of therapy after these events. The 12-month post-treatment follow-up period is represented by the “Treatment completed” state, with a monthly transition probability of 1/12 to the cured state, ensuring the appropriate mean residence time before cure.

The analysis was conducted using a cohort-based Markov model. The model comprised eight mutually exclusive health states: active treatment with BPaL-based regimens containing either moxifloxacin or clofazimine – BPaL(M/C), mSTR, or SOC; alternative individualised second-line (SL) treatment (subsequent regimen) for MDR/RR-TB and pre-XDR-TB; loss to follow-up (LTFU); serious adverse events (SAE); treatment failure (unresolved disease); treatment completion; cure; and death (Fig 1). Transitions between health states occurred in monthly cycles, and the model was run over a 20-year time horizon to capture both immediate and long-term consequences of treatment.

Patients were considered cured if they remained in the treatment completion state for 12 consecutive months without relapse and were alive at the end of this period. Patients entering the cured state were assumed to remain in that state without further TB-related events for the remainder of the model horizon.

### Base-case scenario

Three regimens were compared: BPaL(M/C), mSTR, and SOC. Costs were expressed in 2022 USD, and health outcomes were measured in quality-adjusted life-years (QALYs). Both costs and outcomes were discounted at an annual rate of 3% [5]. For BPaL-based regimens, the treatment duration was 24 weeks. Linezolid dosing was 600 mg daily for the first 16 weeks, followed by 300 mg daily for the remaining 8 weeks, or earlier when moderately tolerated, as implemented under the Belarus national operational research [2], informed by TB-PRACTECAL [6]. Dosing of individual drugs otherwise followed the WHO consolidated guidelines on drug-resistant tuberculosis treatment (2019; 2022 update) [7,8]. The base-case analysis used clinical data from operational research and WHO data to inform transition probabilities, treatment outcomes, and mortality for each strategy (Table 1). The analysis was conducted from a national TB programme perspective.

**Table 1:** Key model input parameters.

Parameter	Value			Source
**Population inputs**				
Gross domestic product per capita (Belarus, 2021), USD	7,489.7			WB [9]
Cohort size: people diagnosed with MDR/RR-TB or pre-XDR-TB (notifications, number of cases)	1,132			WHO [1]
**Regimen-specific treatment outcomes (%)**				
**Parameter**	**BPaL(M/C)**	**mSTR**	**SOC**	**Source**
Successful treatment	93.0	89.81	76.57	OR data
Treatment failure	0.98	3.15	4.18	OR data
Death during treatment	2.10	3.89	7.95	OR data
Lost to follow-up	3.92	3.15	11.30	OR data
Serious adverse events	11.50	16.0	28.03	OR data
Relapse during 12-month post-treatment follow-up ^a^	1.20	0.62	1.64	OR data
Death during 12-month post-treatment follow-up ^a^	4.67	4.95	4.37	OR data
**Serious adverse event outcomes (% among patients with SAE)** ^b^				
**Outcome after SAE**	**BPaL(M/C)**	**mSTR**	**SOC**	**Source**
Continuation of treatment after SAE resolution	78.31	67.05	64.18	OR data
Switched to alternative regimen	3.61	3.41	0	OR data
Treatment failure (unresolved SAE)	0	5.68	7.46	OR data
Death	18.07	23.86	28.36	OR data
**Parameters for SL treatment and alternative subsequent SL regimens in the conservative scenario; informed by MDR-TB and XDR-TB data for Belarus, 2019**				
**Parameter**	MDR-TB		XDR-TB	**Source**
Successful treatment	82.90		71.78	WHO [1]
Treatment failure	1.68		6.62	WHO [1]
Death during treatment	8.29		8.36	WHO [1]
Lost to follow-up	7.12		13.24	WHO [1]
**Health-state utility weights**				
**Health state**	**Utility**			**Source**
On treatment	0.81			Jit [10]
Treatment completed / cured	0.81			Jit [10]
Lost to follow-up / treatment failure / SAE	0.68			Jit [10]
Subsequent SL treatment	0.79			Allel [11]
Death	0			NA
**Average per-patient treatment costs (2022 USD)**				
**Regimen**	**Cost**			**Source**
BPaL(M/C)	6,896			OR data
mSTR	7,681			OR data
SOC	16,899			OR data
**Planned treatment duration**				
**Regimen**	**Duration**			**Source**
BPaL(M/C), days	168			OR data
mSTR, days	273			OR data
SOC, days	560			OR data

BPaL(M/C) treatment regimen containing bedaquiline, pretomanid, linezolid, and moxifloxacin or clofazimine; LTFU lost to follow-up; MDR/RR-TB multidrug-/rifampicin-resistant tuberculosis; mSTR modified 9-month short treatment regimen; OR operational research; pre-XDR-TB pre-extensively drug-resistant tuberculosis; SAE serious adverse event; SL second-line; SOC standard of care; XDR-TB extensively drug-resistant TB; WB World Bank.

^a^Relapse and death during post-treatment follow-up were estimated among patients who successfully completed treatment and were followed for 12 months after treatment completion. ^b^ Adverse event–related outcomes represent mutually exclusive outcomes among patients experiencing a serious adverse event. Serious adverse event (SAE) probabilities were estimated as the proportion of patients with at least one SAE. Probabilities of SAE outcomes (continuation of treatment, switch to an alternative regimen, treatment failure, or death) were estimated among patients with at least one SAE using patient-level data.

Utility weights for each health state were derived from published literature on drug-resistant TB [10]. The monthly probability of LTFU or treatment failure progressing to death was set at 6.86% [12], and the monthly probability of reinitiation of an alternative SL treatment after LTFU was set at 2.3% [13].

Unit costs and resource use were categorised into drugs, inpatient care, outpatient visits, observation, and other costs. S1 Table summarises the distribution of average per-patient treatment costs across regimens, which were used as input parameters in the model. The composition and costs of SOC comparator regimens are described in S2 Table.

### Outcome measures

We estimated incremental costs, incremental QALYs, and incremental net monetary benefit (INMB). Incremental cost-effectiveness ratios (ICERs) were calculated for descriptive purposes where applicable. The willingness-to-pay (WTP) threshold was set at 6,102 USD per QALY, corresponding to 0.81 × gross domestic product (GDP) per capita in Belarus in 2021 (USD 7,489.7), consistent with the approach proposed by Woods et al. [14] for country-specific cost-effectiveness thresholds (0.5–1.0 × GDP per capita). This choice reflects the most recent GDP data available at the time of treatment cost estimation (2022).

### Univariate sensitivity analyses

We performed univariate sensitivity analyses by varying key model parameters across prespecified ranges while holding all other parameters constant. For selected parameters, incremental costs, incremental QALYs, and the resulting INMB were recalculated at the lower and upper bounds, as applicable.

The INMB analysis focused on regimen costs, alternative SL treatment costs, probabilities of relapse after treatment completion, SAE rates, mortality among patients LTFU, and utility parameters. Analyses of incremental costs and QALYs additionally included non-drug costs and the discount rate. Most cost and probability parameters varied by ±25%, and the discount rate varied from 0% to 6%. Specifically, we examined (i) utility after successful treatment (varied by +0.04/ − 0.04), (ii) an aggregated utility for favourable health states (on-treatment, treatment completed, cured; varied by +0.04/ − 0.04), and (iii) an aggregated utility for adverse health states (unresolved disease, SAE, and LTFU; varied by +0.14/ − 0.14) to identify the key drivers of cost-effectiveness outcomes.

### Probabilistic sensitivity analysis

To capture joint parameter uncertainty, we conducted a probabilistic sensitivity analysis using 1,000 Monte Carlo simulations. In each simulation, all uncertain input parameters were sampled from their prespecified probability distributions (S3 Table). Utility weights were sampled from beta distributions. Cost parameters were sampled from gamma distributions, assuming a fixed coefficient of variation of 20% (shape parameter = 25). Sets of mutually exclusive transition probabilities were jointly sampled using Dirichlet distributions to ensure that probabilities from each health state summed to one [15].

For each simulation, total costs and QALYs were recalculated for all strategies. Incremental differences were used to calculate INMB at a WTP threshold of 6,102 USD per QALY. Results were summarised using incremental cost-effectiveness planes, distributions of INMB across simulations and across WTP thresholds, and probabilities of cost-effectiveness (INMB > 0).

### Conservative scenario analysis

To test the robustness of our results under highly restrictive assumptions, we conducted a scenario analysis in which the clinical effectiveness of all regimens was assumed to be identical. Thus, any difference in cost-effectiveness was driven exclusively by treatment duration and cost. This scenario tested whether BPaL(M/C) and mSTR would remain cost-saving even in the absence of differences in treatment outcomes.

### Costs at scale

To estimate the impact on the TB programme budget of introducing shorter regimens for MDR/RR-TB and pre-XDR-TB in Belarus, we conducted a scale-up cost analysis. The analysis used a fixed cohort of 1,132 patients, reflecting the national cohort of patients who started treatment for drug-resistant TB in 2019, thereby standardising comparisons across scenarios. Regimen distributions were derived from operational research data (2020, 2023, and 2024) and the latest national implementation plan. As a reference point, we included a baseline scenario in which all patients received SOC. Total programme cost was estimated for each scenario and presented both in absolute USD and as a percentage of the national TB programme budget.

### Statistical analysis

All analyses were conducted in R version 4.4.1 (R Foundation for Statistical Computing, Vienna, Austria) using RStudio 2023.06.0 + 421. The analysis used a previously published cohort-based Markov framework [11], which was substantially modified and re-parameterised for the present study to incorporate BPaL(M/C), update effectiveness and safety inputs using operational research data, extend the target population to include both MDR/RR-TB and pre-XDR-TB patients, expand the economic outputs and sensitivity analyses (including INMB-based analyses), and extend the scale-up analysis to evaluate the programmatic budget impact of wider adoption of BPaL(M/C) alongside mSTR.

For the base-case analysis, expected costs and QALYs are reported as mean values. Clinical outcomes, including the expected proportion of patients successfully treated and other key outcomes, are presented with 95% confidence intervals (CIs), calculated using the Wilson method.

For probabilistic analysis, total and incremental costs, QALYs, and INMB are reported as mean values with interquartile ranges (IQRs). Probabilistic results are additionally presented graphically to characterise uncertainty in cost-effectiveness outcomes.

This study was a secondary analysis of aggregated operational data. No patients were directly enrolled, and no individual-level identifiable information was used; therefore, ethical approval and informed consent were not required.

The study was reported in accordance with the Consolidated Health Economic Evaluation Reporting Standards 2022 (CHEERS 2022) statement, with the completed checklist available in S4 Table [16].

## Results

### Base case

Across strategies, BPaL(M/C) yielded the lowest total costs and the highest QALYs, followed by mSTR, while SOC was the most costly and least effective. Mean costs and QALYs were USD 8,155 and 10.82 for BPaL(M/C), USD 9,197 and 10.36 for mSTR, and USD 20,757 and 8.91 for SOC, respectively. Incremental analysis showed that BPaL(M/C) and mSTR dominate SOC (Table 2).

**Table 2:** Results of base case scenario.

Strategy	Mean Cost, USD	Mean QALYs	Incremental Cost, USD	Incremental QALYs	INMB, USD	ICER (vs SOC)
SOC	20,757.4	8.9096	− (Reference)	− (Reference)	−	−
mSTR	9,197.1	10.3566	−11,560.29	1.4470	+20,390	Dominant
BPaL(M/C)	8,154.6	10.8224	−12,602.77	1.9127	+24,274	Dominant

BPaL(M/C) treatment regimen containing bedaquiline, pretomanid, linezolid, and moxifloxacin or clofazimine; QALYs quality-adjusted life-years; INMB incremental net monetary benefit; mSTR modified 9-month short treatment regimen; ICER incremental cost-effectiveness ratio; SOC standard of care.

These findings are illustrated by the cost-effectiveness plane of expected costs and QALYs (S1 Fig) and the incremental cost-effectiveness plane relative to SOC (S2 Fig), where both BPaL(M/C) and mSTR are located in the cost-effective region.

### Clinical outcomes

The expected proportion of patients successfully treated (cured) was 87.7% (95% CI 85.7–89.5) for BPaL(M/C), 83.3% (81.0–85.4) for mSTR, and 68.3% (65.5–70.9) for SOC.

Cumulative mortality over the 20-year horizon – comprising deaths during treatment, deaths during the 12-month post-treatment follow-up period, and deaths following LTFU or treatment failure – was substantially lower for shorter regimens: 12.3% (95% CI 10.5–14.3) for BPaL(M/C), 16.7% (95% CI 14.6–19.0) for mSTR, and 31.7% (95% CI 29.1–34.5) for SOC.

**Fig 2:**
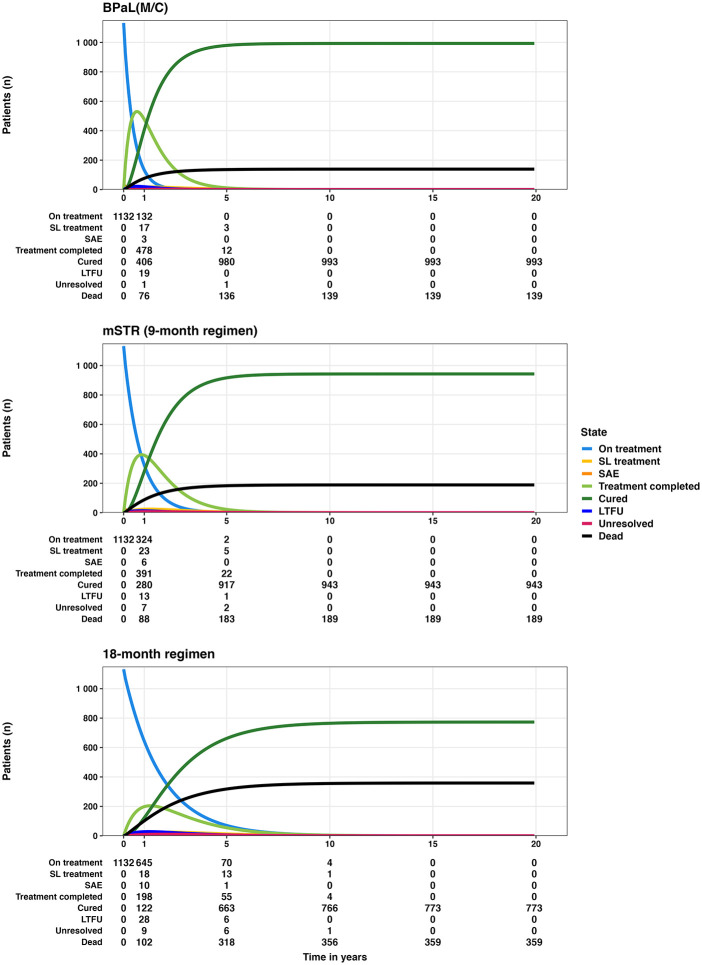
Modelled cohort trajectories across health states over a 20-year horizon. Panels show the expected number of patients occupying each health state over time for BPaL(M/C), mSTR, and the 18-month regimen (SOC), assuming an initial cohort of 1,132 patients.

Modelled cohort trajectories showed that most transitions between health states occurred within the first years after treatment initiation, with outcomes stabilising thereafter, indicating that the 20-year time horizon was sufficient to capture long-term consequences (Fig 2).

Over the long-term horizon, the proportions remaining in LTFU or treatment failure states became negligible, such that cumulative mortality represented the primary unfavourable clinical outcome in the model (S5 Table).

### Univariate sensitivity analyses

The univariate sensitivity analyses showed that non-drug costs (particularly for SOC and mSTR) and the discount rate had the greatest influence on incremental costs (S3 Fig, S6 Table). In contrast, incremental costs for the comparison between BPaL(M/C) and mSTR were substantially less sensitive to parameter variation.

Assumptions regarding the utility of the favourable health state and discounting had the strongest impact on incremental QALYs (S4 Fig, S6 Table). Variations in these parameters affected absolute QALY gains, but did not change the relative ranking of strategies.

**Fig 3:**
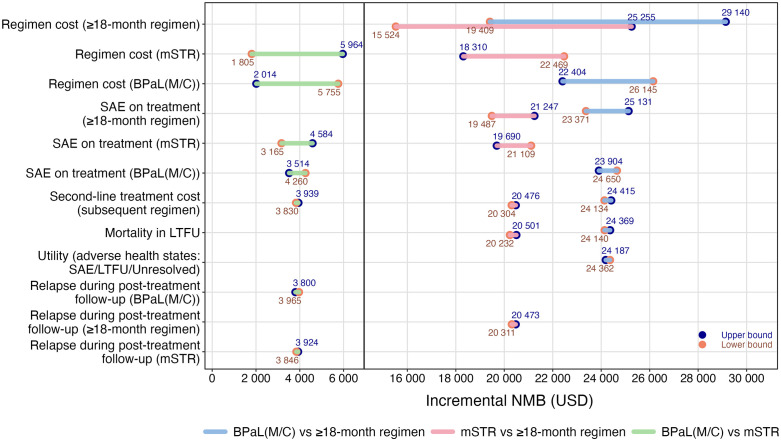
Determinants of incremental net monetary benefit: univariate sensitivity analysis. Tornado plot showing the seven parameters with the largest impact on incremental net monetary benefit (INMB) for each pairwise comparison. Full values for all parameters are provided in S7 Table. Upper and lower bounds represent the ranges of parameters explored in the univariate sensitivity analysis. BPaL(M/C) treatment regimen containing bedaquiline, pretomanid, linezolid, and moxifloxacin or clofazimine, NMB net monetary benefit, LTFU lost to follow-up, mSTR modified 9-month short treatment regimen, SAE serious adverse event.

Across all comparisons, regimen costs were the dominant drivers of INMB (Fig 3, S7 Table). Changes in SAE rates had a modest effect, whereas relapse probabilities, alternative SL treatment costs, utilities for adverse health states, utility after successful treatment, and mortality among patients LTFU had minimal influence on INMB.

The results of the univariate sensitivity analysis were consistent with the base case findings, confirming that BPaL(M/C) remained the most cost-effective strategy across plausible parameter ranges.

### Probabilistic sensitivity analysis

**Fig 4:**
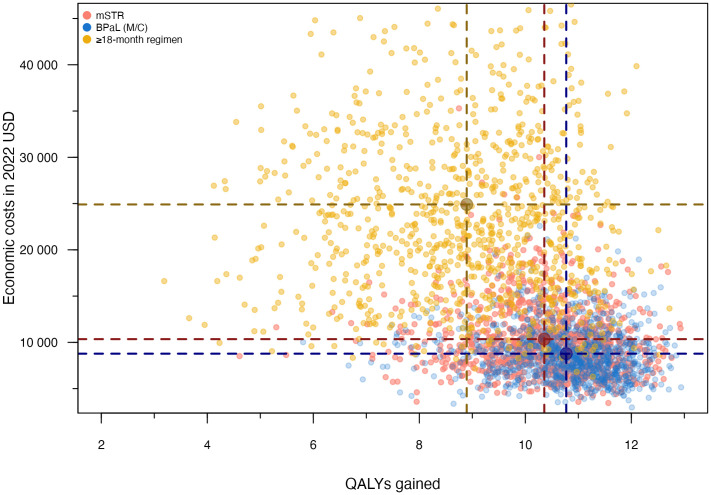
Probabilistic sensitivity analysis: impacts on economic costs and QALYs per treatment arm. BPaL(M/C) treatment regimen containing bedaquiline, pretomanid, linezolid, and moxifloxacin or clofazimine; mSTR modified 9-month short treatment regimen; QALYs quality-adjusted life-years.

Across simulations, BPaL(M/C) consistently clustered in the lower-cost, higher-QALYs region of the cost-effectiveness plane, mSTR occupied an intermediate position, and SOC was associated with the highest costs and lowest QALYs (Fig 4). The mean [IQR] total costs and QALYs were USD 8,777 [6,807; 10,201] and 10.77 [10.20; 11.49] QALYs for BPaL(M/C), USD 10,348 [7,747; 12,049] and 10.36 [9.69; 11.17] QALYs for mSTR, and USD 24,905 [16,769; 30,161] and 8.89 [7.77; 10.22] QALYs for SOC.

**Fig 5:**
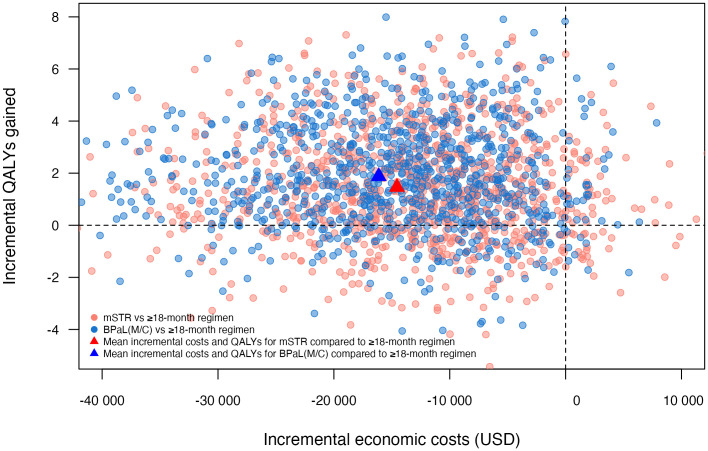
Incremental cost-effectiveness planes: probabilistic sensitivity analysis. Points represent simulated incremental costs and QALYs relative to the ≥ 18-month regimen. Triangles indicate mean incremental costs and QALYs for each shorter regimen. BPaL(M/C) treatment regimen containing bedaquiline, pretomanid, linezolid, and moxifloxacin or clofazimine; mSTR modified 9-month short treatment regimen; QALYs quality-adjusted life-years.

Pairwise incremental analyses showed dominance of both BPaL(M/C) and mSTR over SOC (Fig 5 and S5 Fig). For BPaL(M/C) versus SOC, the mean [IQR] incremental cost was USD −16,127 [−21,575; −8,083] with a mean [IQR] QALY gain of 1.88 [0.51; 3.20], while for mSTR versus SOC the corresponding values were USD −14,557 [−20,157; −6,397] and 1.46 [0.05; 2.80] QALYs, respectively.

**Fig 6:**
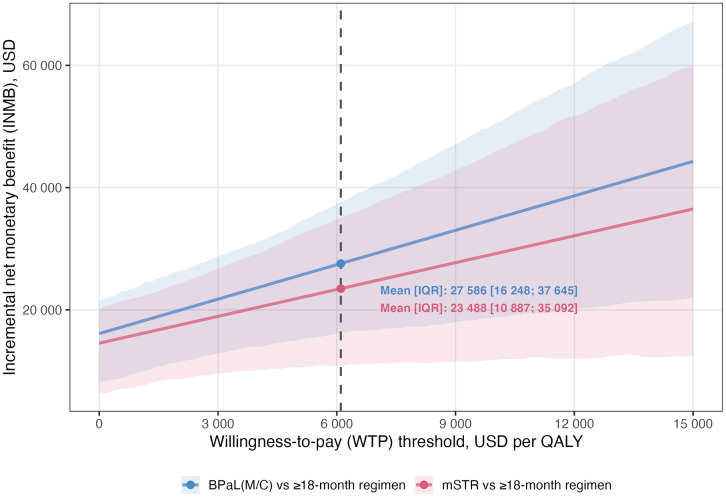
Incremental net monetary benefit (INMB) across WTP thresholds. BPaL(M/C) treatment regimen containing bedaquiline, pretomanid, linezolid, and moxifloxacin or clofazimine; INMB incremental net monetary benefit; IQR interquartile range; mSTR modified 9-month short treatment regimen; QALY quality-adjusted life-year; WTP willingness-to-pay.

Analysis of incremental net monetary benefit across WTP thresholds (Fig 6) indicated a strong economic preference for both BPaL(M/C) and mSTR over SOC. At the Belarus-specific threshold, the mean [IQR] INMB was USD 27,586 [16,248; 37,645] for BPaL(M/C) versus SOC and USD 23,488 [10,887; 35,092] for mSTR versus SOC.

The probability of cost-effectiveness across willingness-to-pay thresholds was consistent with the base-case findings and served as a descriptive measure of decision uncertainty, to be interpreted alongside expected value-based outcomes (S6 Fig).

### Conservative scenario

In the conservative scenario, where clinical effectiveness was assumed to be identical across regimens, both BPaL(M/C) and mSTR remained cost-saving compared with SOC, although they generated slightly fewer QALYs (Table 3). These findings indicate that the economic advantage of shorter regimens persists even in the absence of differences in treatment outcomes and is primarily driven by reduced treatment duration and lower costs.

**Table 3:** Results of conservative scenario.

Strategy	Mean Cost, USD	Mean QALYs	Incremental Cost, USD	Incremental QALYs	INMB, USD	ICER (vs SOC)
All patients						
SOC	21,189.0	9.5205	− (Reference)	− (Reference)	−	−
mSTR	10,428.9	9.3400	−10,760.1	−0.1805	+9,659	Cost-saving
BPaL(M/C)	9,669.5	9.2671	−11,519.5	−0.2534	+9,973	Cost-saving
Patients with treatment success only						
SOC	28,579.8	12.8412	− (Reference)	− (Reference)	−	−
mSTR	14,065.7	12.5970	−14,514.1	−0.2442	+13,024	Cost-saving
BPaL(M/C)	13,041.4	12.4987	−15,538.4	−0.3426	+13,448	Cost-saving

BPaL(M/C) treatment regimen containing bedaquiline, pretomanid, linezolid, and moxifloxacin or clofazimine; QALYs quality-adjusted life-years; INMB incremental net monetary benefit; ICER incremental cost-effectiveness ratio; mSTR modified 9-month short treatment regimen; SOC standard of care.

### Costs at scale

In the scale-up analysis (Table 4), total treatment costs varied substantially according to the distribution of regimens. If all patients were treated with SOC, total costs were estimated at USD 19.1 million, corresponding to 25.8% of the national TB programme budget. Introduction of shorter regimens resulted in marked cost reductions. Under observed regimen distributions in 2020 (25.7% mSTR and 2.3% BPaL(M/C)), projected costs declined to USD 16.2 million (21.8% of the budget). With wider adoption of BPaL(M/C) by 2024, total costs decreased further to USD 9.5 million (12.8%). According to the current national implementation plan, assuming 77.5% BPaL(M/C), 10% mSTR and 12.5% SOC, expenditures are projected to reach USD 9.3 million, representing 12.6% of the national TB programme budget.

**Table 4:** Results of scale-up cost analysis.

People diagnosed with MDR/RR-TB / pre-XDR-TB (notifications, number of cases, 2020) [1]	Total NTP budget 2020, available funding in USD (domestic + global fund + other grants) [1]	Percentage of people started mSTR for RR/MDR- TB / pre-XDR-TB	Percentage of people started BPaL(M/C) for RR/MDR-TB / pre-XDR-TB	Percentage of people started SOC for MDR/RR- TB / pre-XDR-TB	Average cost savings at scale (SOC total costs minus costs under mixed SOC / mSTR / BPaL(M/C) scenarios)	Total costs at scale (average cost per mSTR / BPaL(M/C) regimen × total number of patients receiving mSTR / BPaL(M/C) + average SOC cost × total number of patients ineligible for mSTR / BPaL(M/C)), thousand USD	Percentage of costs related to NTP budget (i.e., % of cost at scale if MDR/RR- TB/ pre-XDR-TB patients are treated with respective mixed SOC /mSTR/ BPaL(M/C) regimens divided by total NTP budget)
1,132	74,205,441	0%	0%	100%	0	19,129,668	25.8%
		25.7%	2.3%	72.0%	2,942,225	16,187,443	21.8%
		27.6%	25.7%	46.7%	5,786,577	13,343,091	18.0%
		22.0%	64.5%	13.5%	9,597,223	9,532,445	12.8%
		10.0%	77.5%	12.5%	9,814,152	9,315,516	12.6%

BPaL(M/C) treatment regimen containing bedaquiline, pretomanid, linezolid, and moxifloxacin or clofazimine; MDR/RR-TB multidrug-/rifampicin-resistant tuberculosis; NTP national tuberculosis programme; mSTR modified 9-month short treatment regimen; pre-XDR-TB pre-extensively drug-resistant tuberculosis; SOC standard of care.

## Discussion

This economic evaluation provides new evidence on the cost-effectiveness of BPaL(M/C) and mSTR compared with SOC for MDR/RR-TB and pre-XDR-TB in Belarus. Across probabilistic and deterministic analyses, BPaL(M/C) was the most cost-effective option, combining the lowest total costs with the highest QALYs. mSTR was also cost-effective relative to SOC, although less effective than BPaL(M/C). These findings align with international evidence indicating that shorter, all-oral regimens can substantially reduce both health system costs and patient burden while improving clinical outcomes [17,18].

Consistent with the clinical data used to inform the model, both BPaL(M/C) and mSTR were associated with higher treatment success, low relapse rate following treatment completion, and fewer unfavourable outcomes compared with SOC. The most pronounced improvement was observed in reduced LTFU, likely reflecting better tolerability and adherence under shorter regimens. The economic advantage of BPaL(M/C) and mSTR is therefore largely explained by their shorter duration and better clinical outcomes, both of which ultimately reduce resource use. This explains their dominance over SOC.

Sensitivity analyses further supported the robustness of these findings. Univariate sensitivity analyses identified regimen costs as the primary drivers of INMB. Clinical parameters, including relapse risk, adverse event rates, mortality among patients LTFU, and health state utilities, contributed to uncertainty, but did not alter the relative ranking of regimens within plausible ranges.

Probabilistic sensitivity analysis supported these conclusions, demonstrating high probabilities of cost-effectiveness for both BPaL(M/C) and mSTR compared with SOC at the Belarus-specific WTP threshold. Even under conservative assumptions of identical clinical effectiveness, both shorter regimens remained cost-saving relative to SOC, indicating that their economic advantage is primarily driven by shorter treatment duration and associated reductions in resource use.

The scale-up analysis demonstrated that nationwide introduction of shorter regimens could nearly halve the share of the national TB programme budget devoted to MDR/RR-TB and pre-XDR-TB treatment. Wider implementation of BPaL(M/C) and mSTR in place of conventional ≥18-month regimens was projected to reduce this budget share from 25.8% to approximately 12.6%, corresponding to annual savings exceeding USD 9.3 million. Such savings could be reallocated to strengthen diagnostic capacity, expand patient support, and strengthen the overall TB programme. Thus, the economic benefit of shorter regimens extends beyond patient-level cost-effectiveness to the programme level, supporting large-scale implementation that is clinically justified and financially sustainable.

Our findings are consistent with a growing body of international economic evidence supporting shorter all-oral regimens for drug-resistant TB. Early modelling studies suggested that BPaL-based regimens could be cost-effective or cost-saving compared with conventional long treatment regimens, using programme cost data for standard care and trial-based efficacy inputs for BPaL-based regimens [17].

Subsequent analyses incorporating TB-PRACTECAL data extended these findings across multiple high-burden settings, demonstrating reductions in costs with improved health outcomes versus standard care [19]. In parallel, Mulder et al. [20] modelled the phased introduction of BPaL in Indonesia, Kyrgyzstan, and Nigeria and projected substantial budgetary savings, highlighting the potential economic benefits of BPaL implementation in settings with a high burden of drug-resistant TB. More broadly, Nagar et al. [21] emphasised the growing role of economic modelling and regimen shortening as key strategies for improving efficiency and resource allocation in TB control.

Most recently, trial-based cost–utility analyses alongside TB-PRACTECAL confirmed that BPaL-based regimens reduce costs and improve health outcomes [4]. Collectively, these studies establish the potential value of shorter regimens but are often based on trial efficacy or focus on single regimens. The present study complements and extends this evidence in three important ways: first, by drawing on detailed operational data from Belarus, including regimen-specific costs and treatment pathways, to reflect real-world implementation; second, by evaluating both BPaL(M/C) and mSTR within a single comparative framework against SOC and against each other – an area that remains underexplored in the international literature; and third, by linking patient-level cost-effectiveness with a national scale-up analysis to quantify the budgetary implications of regimen choice for the Belarus TB programme.

This study has several strengths. First, it draws on country-specific programmatic data from Belarus on clinical outcomes, costs, and patient flows, providing a realistic representation of routine practice rather than relying solely on trial-based or secondary sources. Second, we jointly evaluated BPaL(M/C) and mSTR against SOC, enabling both direct comparisons with standard care and head-to-head assessment between novel regimens. Third, the analysis integrated patient-level cost-effectiveness with a programmatic scale-up assessment, linking incremental outcomes to national budgetary impact. Finally, the use of multiple sensitivity analyses – including univariate, probabilistic, and conservative scenarios – enhanced confidence in the robustness of findings across a wide range of assumptions.

Several limitations should also be acknowledged. Utility weights were derived from published literature, which may not fully capture health-related quality of life in the Belarus setting. Two transition probabilities were taken from the literature: (i) progression from LTFU or treatment failure to death, and (ii) reinitiation of SL treatment after LTFU. Finally, costs for managing SAE were approximated using average monthly non-drug treatment costs, which may not fully reflect the resource use associated with specific adverse events. Despite these limitations, the consistency of results across multiple analytic approaches suggests that the main conclusions are robust.

Taken together, these findings indicate that BPaL(M/C) and mSTR are not only cost-effective at the patient level but also financially sustainable at the programmatic scale. This provides a strong rationale for their scale-up in Belarus.

From a policy perspective, key priorities include obtaining acceptable prices for medicines and diagnostics, ensuring effective and sustained implementation of shorter regimens, and ongoing monitoring of their effectiveness in real-world settings. Cost savings should be reinvested in expanding diagnostic capacity, supporting patients, and strengthening the programme.

## Supporting information

S1 Fig Cost-effectiveness plane of expected costs and QALYs for MDR/RR-TB and pre-XDR-TB treatment strategies. (TIFF)

S2 Fig Incremental cost-effectiveness plane relative to SOC. (TIFF)

S3 Fig Univariate sensitivity analyses – impact on incremental economic costs. Upper and lower bounds represent the ranges of parameters explored in the univariate sensitivity analysis.
(TIFF)

S4 Fig Univariate sensitivity analyses – impact on incremental QALYs. Upper and lower bounds represent the ranges of parameters explored in the univariate sensitivity analysis.
(TIFF)

S5 Fig Distribution of probabilistic sensitivity analysis simulations across incremental cost–effectiveness outcomes. (TIFF)

S6 Fig Probability of cost-effectiveness (INMB > 0) across willingness-to-pay thresholds. This figure is presented as a descriptive measure of decision uncertainty and should be interpreted alongside expected value-based outcomes.
(TIFF)

S1 Table Distribution of average per-patient treatment costs across regimens in 2022 USD. (DOCX)

S2 Table Standard of care comparator regimens. (DOCX)

S3 Table Probability distributions, parameterisation, and uncertainty ranges assigned to model inputs for probabilistic sensitivity analysis. (DOCX)

S4 Table CHEERS 2022 checklist. (DOCX)

S5 Table Expected clinical outcomes over a 20-year horizon (per 1,132 patients). (DOCX)

S6 Table Univariate sensitivity analyses – impact on incremental costs, QALYs, and INMB. (DOCX)

S7 Table Univariate sensitivity analyses – impact on incremental net monetary benefit (INMB). (DOCX)
