# Supplementary figures and images for: Cost-effectiveness of BPaL-based and 9-month modified all-oral short treatment regimens for rifampicin-resistant tuberculosis in Belarus
Source: PLOS Glob Public Health. 2026 Jul 23;6(7):e0005872. doi: 10.1371/journal.pgph.0005872 (PMC13395433; doi:10.1371/journal.pgph.0005872)

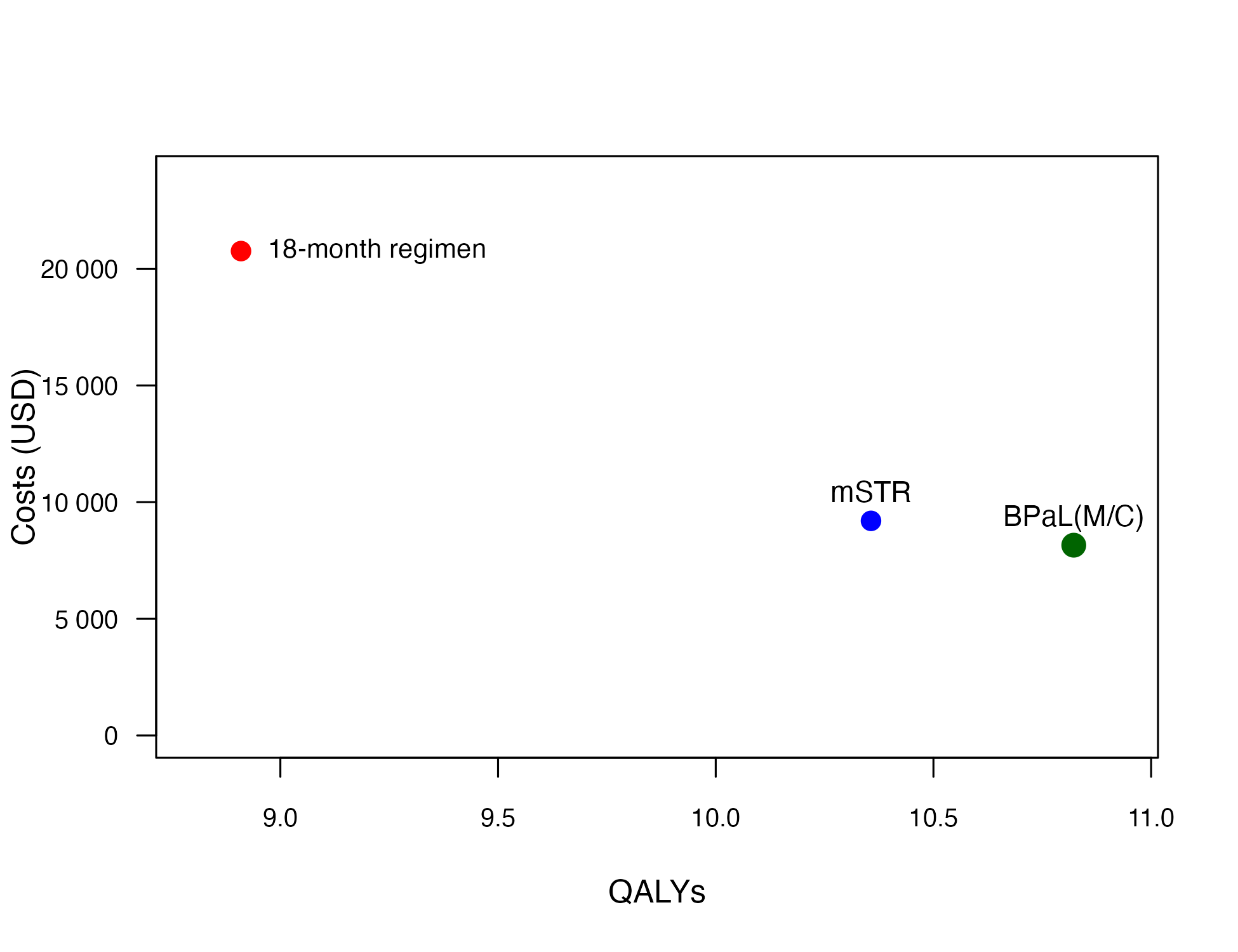

Supplement: S1 Fig — (TIFF) [file pgph.0005872.s001.tiff]

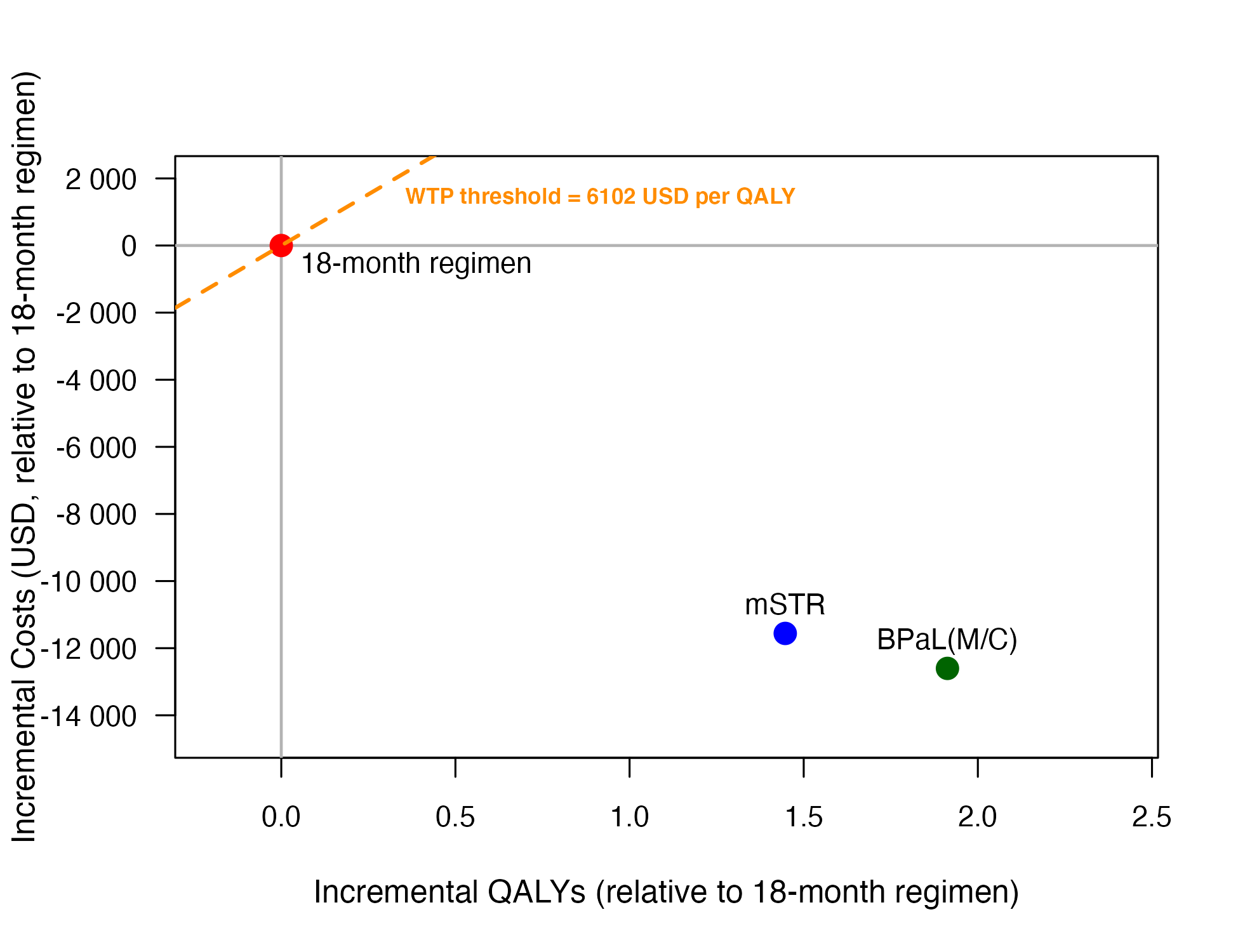

Supplement: S2 Fig — (TIFF) [file pgph.0005872.s002.tiff]

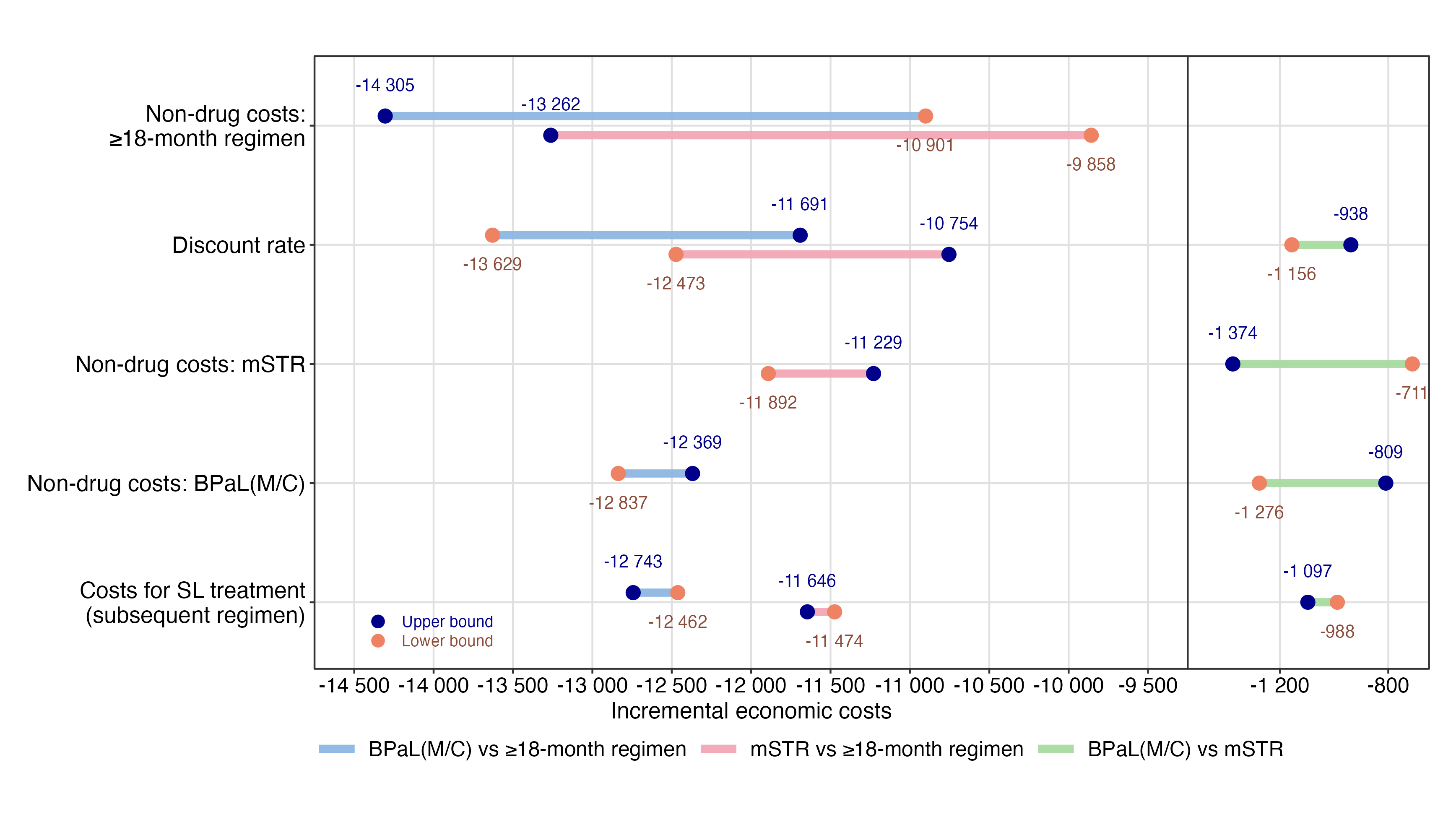

Supplement: S3 Fig — Upper and lower bounds represent the ranges of parameters explored in the univariate sensitivity analysis. (TIFF) [file pgph.0005872.s003.tiff]

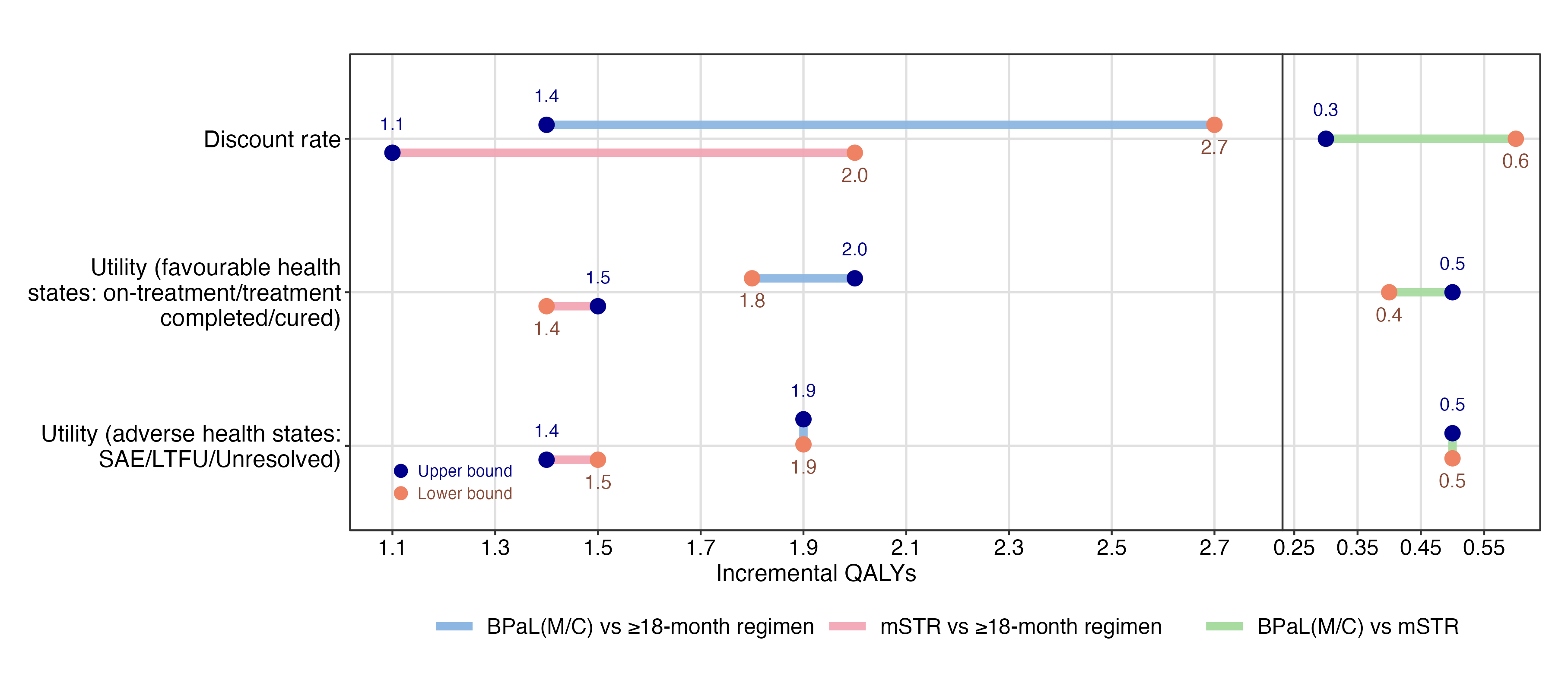

Supplement: S4 Fig — Upper and lower bounds represent the ranges of parameters explored in the univariate sensitivity analysis. (TIFF) [file pgph.0005872.s004.tiff]

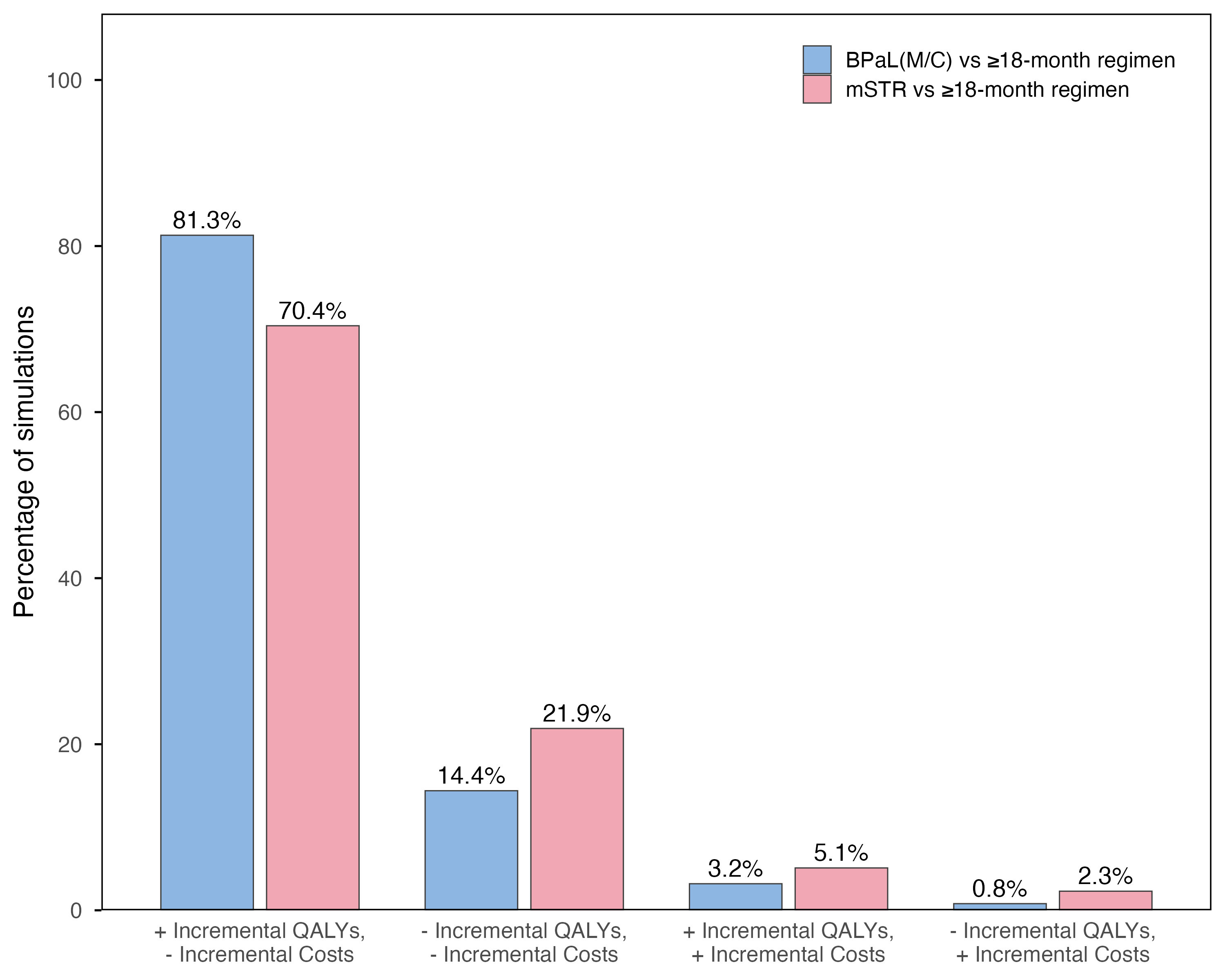

Supplement: S5 Fig — (TIFF) [file pgph.0005872.s005.tiff]

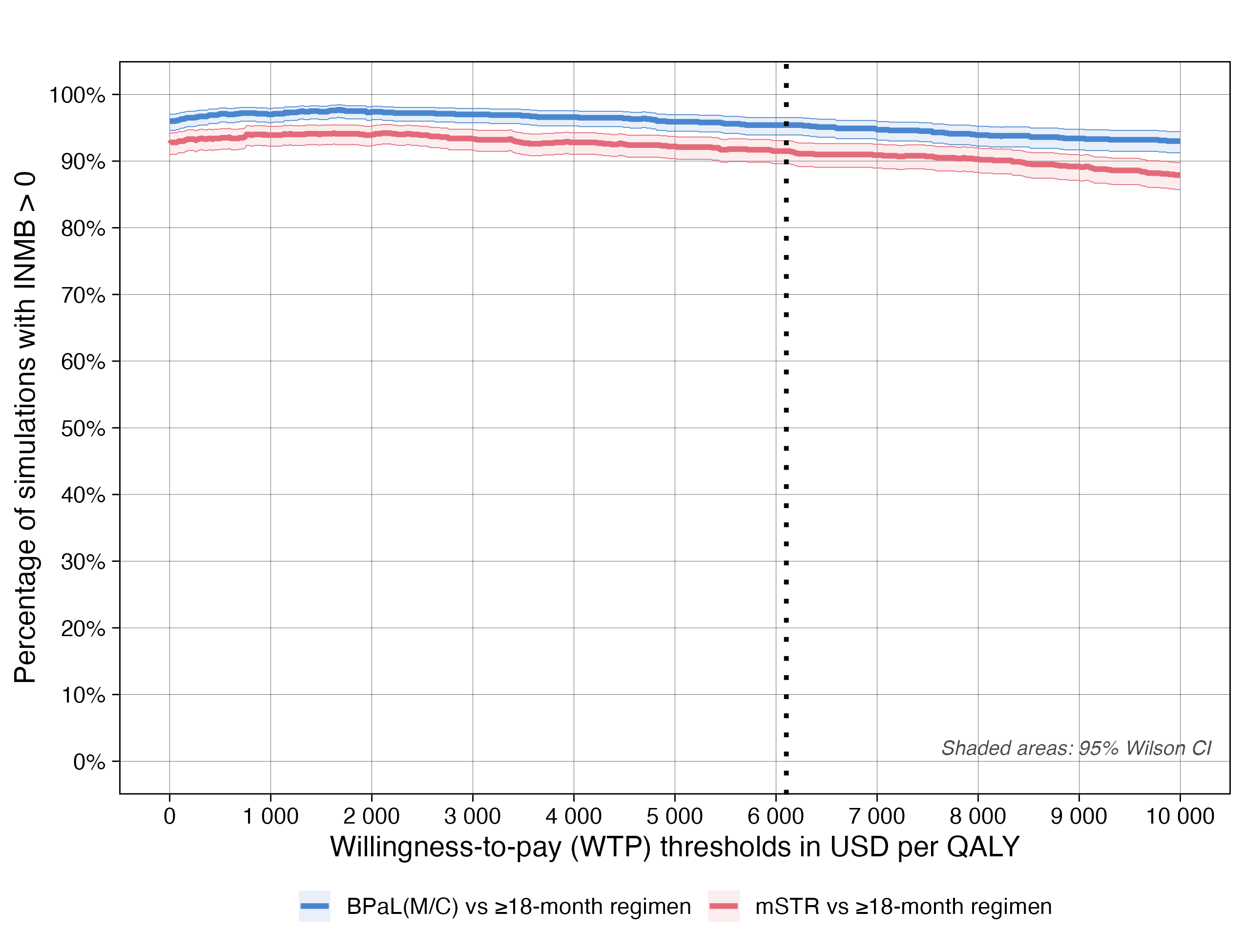

Supplement: S6 Fig — This figure is presented as a descriptive measure of decision uncertainty and should be interpreted alongside expected value-based outcomes. (TIFF) [file pgph.0005872.s006.tiff]
