# Supplementary material for: Cost-effectiveness of BPaL-based and 9-month modified all-oral short treatment regimens for rifampicin-resistant tuberculosis in Belarus
Source: PLOS Glob Public Health. 2026 Jul 23;6(7):e0005872. doi: 10.1371/journal.pgph.0005872 (PMC13395433; doi:10.1371/journal.pgph.0005872)
Supplement: S1 Table — (DOCX) [file pgph.0005872.s007.docx]

**S1 Table. Distribution of average per-patient treatment costs across regimens in 2022 USD^a^.**

| Cost component | Description | Regimen | | |
| --- | --- | --- | --- | --- |
|  |  | BPaL(M/C) | mSTR | SOC |
| Drugs | Average drug regimen cost (full course) | 862 | 1,225 | 5,912 |
| Inpatient | Average number of inpatient days for treatment initiation × Cost per inpatient day | 5,259 | 5,259 | 8,764 |
| Outpatient | Number of outpatient visits × Costs per outpatient visit | 133 | 248 | 382 |
| Monitoring | Monitoring costs per patient | 434 | 508 | 780 |
| Observation | (Average number of doses observed by video × Cost per dose observed by video) + (Average number of doses directly observed × Cost per dose directly observed) | 68 | 161 | 361 |
| Other | Average psychosocial support costs + Average home-based care costs | 140 | 280 | 700 |
| Total | | **6,896** | **7,681** | **16,899** |

^a^ 1 USD = 2.63 Belarusian rubles.
