# Supplementary material for: Cost-effectiveness of BPaL-based and 9-month modified all-oral short treatment regimens for rifampicin-resistant tuberculosis in Belarus
Source: PLOS Glob Public Health. 2026 Jul 23;6(7):e0005872. doi: 10.1371/journal.pgph.0005872 (PMC13395433; doi:10.1371/journal.pgph.0005872)
Supplement: S2 Table — (DOCX) [file pgph.0005872.s008.docx]

**S2 Table. Standard of care comparator regimens.**

| Comparator regimens^a^ | Regimen cost, USD | Number of patients, initiated treatment, 2020-2022 |
| --- | --- | --- |
| **MDR/RR-TB** | | |
| 6 Bdq Mfx Lzd Cfz Cs/14Mfx Lzd Cfz Cs | 2,606 | 2 |
| 18 Bdq Lfx Lzd Cfz Cs | 3,145 | 484 |
| 20 Bdq Mfx Lzd Cfz Cs | 3,214 | 31 |
| **pre-XDR-TB** | | |
| 20 Bdq Lzd Cfz Cs Dlm | 8,254 | 338 |
| 6-8 Bdq Lzd Cfz Cs Imp/Amx/Clv / 14-12 Bdq Lzd Cfz Cs | 5,921 | 29 |
| 6-8 Bdq Lzd Cfz Cs Dlm Imp/Amx/Clv /14-12 Bdq Lzd Cfz Cs Dlm | 11,588 | 12 |
| 20 Bdq Lzd Cfz Dlm | 8,139 | 256 |

Amx/Clv amoxicillin/clavulanate; Bdq bedaquiline; Cfz clofazimine; Cs cycloserine; Dlm delamanid; Imp imipenem/cilastatin; Lfx levofloxacin; Lzd linezolid; MDR/RR-TB multidrug-/rifampicin-resistant tuberculosis; Mfx moxifloxacin; Pa pretomanid; pre-XDR-TB pre-extensively drug-resistant tuberculosis.

^a^ Dosing of individual drugs was aligned with WHO consolidated guidelines on drug-resistant tuberculosis treatment (2019; 2022 update) [7,8].
