# Supplementary material for: Cost-effectiveness of BPaL-based and 9-month modified all-oral short treatment regimens for rifampicin-resistant tuberculosis in Belarus
Source: PLOS Glob Public Health. 2026 Jul 23;6(7):e0005872. doi: 10.1371/journal.pgph.0005872 (PMC13395433; doi:10.1371/journal.pgph.0005872)
Supplement: S3 Table — (DOCX) [file pgph.0005872.s009.docx]

**S3 Table. Probability distributions, parameterisation, and uncertainty ranges assigned to model inputs for probabilistic sensitivity analysis.**

| **Variable Name** | **Base-case value** | **SD** | **Distribution** | **Parameters** | **Source for Base-case value** |
| --- | --- | --- | --- | --- | --- |
| **Monthly costs, 2022 USD** |  |  | **Gamma** | **shape (k) = 25, scale (θ) = mean / 25 ^a^** |  |
| **BPaL(M/C)** |  |  |  |  |  |
| Average monthly treatment cost per patient for BPaL(M/C), USD | 1253.82 | 250.76 | Gamma | k = 25, θ = 50.15 | OR data |
| Monthly cost of SAE management for BPaL(M/C) per patient, USD | 1097.09 | 219.42 | Gamma | k = 25, θ = 43.88 | OR data |
| **mSTR** |  |  |  |  |  |
| Average monthly treatment cost per patient for mSTR, USD | 844.07 | 168.81 | Gamma | k = 25, θ = 33.76 | OR data |
| Monthly cost of SAE management for mSTR per patient, USD | 709.47 | 141.89 | Gamma | k = 25, θ = 28.38 | OR data |
| **SOC** |  |  |  |  |  |
| Average monthly treatment cost per patient for SOC, USD | 933.53 | 186.71 | Gamma | k = 25, θ = 37.34 | OR data |
| Monthly cost of SAE management for SOC per patient, USD | 606.94 | 121.39 | Gamma | k = 25, θ = 24.28 | OR data |
| **Common costs** |  |  |  |  |  |
| Average monthly treatment cost per patient for subsequent SL, USD | 933.53 | 186.71 | Gamma | k = 25, θ = 37.34 | OR data |
| Average monthly cost of tracing patients lost to follow-up per patient, USD | 1 | 0.20 | Gamma | k = 25, θ = 0.04 | OR data |
| **Utility weights (annual)** |  |  | **Beta** | **shape parameters (alpha = [Base-case value] x 100, beta = (1 – Base-case value) x 100** |  |
| Utility: RR-TB patients receiving initial treatment | 0.81 | 0.04 | Beta | alpha = 81, beta = 19 | Jit, 2011 [10] |
| Utility: RR-TB patients (SL treatment - subsequent regimens) | 0.79 | 0.04 | Beta | alpha = 79, beta = 21 | Allel, 2025 [11] |
| Utility: Patients lost to follow-up | 0.68 | 0.05 | Beta | alpha = 68, beta = 32 | Jit, 2011 [10] |
| Utility: Patients receiving end-of-life care | 0.68 | 0.05 | Beta | alpha = 68, beta = 32 | Jit, 2011 [10] |
| Utility: TB patients with treatment completed | 0.81 | 0.04 | Beta | alpha = 81, beta = 19 | Jit, 2011 [10] |
| Utility: RR-TB patients with adverse events | 0.68 | 0.05 | Beta | alpha = 68, beta = 32 | Jit, 2011 [10] |
| Utility: TB patients with cured TB | 0.81 | 0.04 | Beta | alpha = 81, beta = 19 | Jit, 2011 [10] |
| **Monthly transition probabilities** |  |  |  |  |  |
| **BPaL(M/C) core transitions** |  |  | **Dirichlet ^b^** |  |  |
| BPaL(M/C) → Lost to follow-up | 0.0063 | 0.0079 | Dirichlet | alpha = 0.631 | OR data |
| BPaL(M/C) → Successful treatment completion | 0.1496 | 0.0355 | Dirichlet | alpha = 14.9648 | OR data |
| BPaL(M/C) → Serious adverse event | 0.0209 | 0.0142 | Dirichlet | alpha = 2.0902 | OR data |
| BPaL(M/C) → Death during treatment | 0.0034 | 0.0058 | Dirichlet | alpha = 0.3381 | OR data |
| BPaL(M/C) → Switch to SL treatment (subsequent regimens) | 0.0016 | 0.0039 | Dirichlet | alpha = 0.1578 | OR data |
| Remaining in BPaL(M/C) state | 0.8182 | 0.0384 | Dirichlet | alpha = 81.8182 | OR data |
| **Serious Adverse Event (BPaL(M/C))** |  |  |  |  |  |
| Serious adverse event → Continuation of BPaL(M/C) after SAE resolution | 0.7831 | 0.0410 | Dirichlet | alpha = 78.3133 | OR data |
| Serious adverse event → Switch to SL treatment (subsequent regimens) after SAE (BPaL(M/C)) | 0.0361 | 0.0186 | Dirichlet | alpha = 3.6145 | OR data |
| Serious adverse event → Death (BPaL(M/C)) | 0.1807 | 0.0383 | Dirichlet | alpha = 18.0723 | OR data |
| **Treatment completed transitions (BPaL(M/C))** |  |  |  |  |  |
| Treatment completed → Relapse (BPaL(M/C)) | 0.0010 | 0.0032 | Dirichlet | alpha = 0.1004 | OR data |
| Treatment completed → Long-term cure (after 12 months) (BPaL(M/C)) | 0.0829 | 0.0274 | Dirichlet | alpha = 8.2925 | OR data |
| Treatment completed → Death (BPaL(M/C)) | 0.0039 | 0.0062 | Dirichlet | alpha = 0.3891 | OR data |
| Remaining in Treatment completed state (BPaL(M/C)) | 0.9122 | 0.0282 | Dirichlet | alpha = 91.218 | OR data |
| **mSTR core transitions** |  |  |  |  |  |
| mSTR → Lost to follow-up | 0.0029 | 0.0054 | Dirichlet | alpha = 0.2938 | OR data |
| mSTR → Successful treatment completion | 0.0838 | 0.0276 | Dirichlet | alpha = 8.3827 | OR data |
| mSTR → Serious adverse event | 0.0178 | 0.0131 | Dirichlet | alpha = 1.7778 | OR data |
| mSTR → Death during treatment | 0.0036 | 0.0060 | Dirichlet | alpha = 0.363 | OR data |
| mSTR → Switch to SL treatment (subsequent regimens) | 0.0029 | 0.0054 | Dirichlet | alpha = 0.2938 | OR data |
| Remaining in mSTR state | 0.8889 | 0.0313 | Dirichlet | alpha = 88.8889 | OR data |
| **Serious Adverse Event (mSTR)** |  |  |  |  |  |
| Serious adverse event → Continuation of mSTR after SAE resolution | 0.6705 | 0.0468 | Dirichlet | alpha = 67.0455 | OR data |
| Serious adverse event → Switch to SL treatment (subsequent regimens) after SAE (mSTR) | 0.0341 | 0.0181 | Dirichlet | alpha = 3.4091 | OR data |
| Serious adverse event → Death (mSTR) | 0.2386 | 0.0424 | Dirichlet | alpha = 23.8636 | OR data |
| Serious adverse event → Treatment failure (unresolved) (mSTR) | 0.0568 | 0.0230 | Dirichlet | alpha = 5.6818 | OR data |
| **Treatment completed (mSTR)** |  |  |  |  |  |
| Treatment completed → Relapse (mSTR) | 0.0005 | 0.0023 | Dirichlet | alpha = 0.0515 | OR data |
| Treatment completed → Long-term cure (after 12 months) (mSTR) | 0.0829 | 0.0274 | Dirichlet | alpha = 8.2947 |  |
| Treatment completed → Death (mSTR) | 0.0041 | 0.0064 | Dirichlet | alpha = 0.4124 | OR data |
| Remaining in Treatment completed state (mSTR) | 0.9124 | 0.0281 | Dirichlet | alpha = 91.2414 | OR data |
| **SOC core transitions** |  |  |  |  |  |
| SOC → Lost to follow-up | 0.0045 | 0.0067 | Dirichlet | alpha = 0.4517 | OR data |
| SOC → Successful treatment completion | 0.0306 | 0.0171 | Dirichlet | alpha = 3.0613 | OR data |
| SOC → Serious adverse event | 0.0156 | 0.0123 | Dirichlet | alpha = 1.5574 | OR data |
| SOC → Death during treatment | 0.0032 | 0.0056 | Dirichlet | alpha = 0.3178 | OR data |
| SOC → Switch to SL treatment (subsequent regimens) | 0.0017 | 0.0041 | Dirichlet | alpha = 0.1673 | OR data |
| Remaining in SOC state | 0.9444 | 0.0228 | Dirichlet | alpha = 94.4444 | OR data |
| **Serious Adverse Event SOC** |  |  |  |  |  |
| Serious adverse event → Continuation of SOC after SAE resolution | 0.6418 | 0.0477 | Dirichlet | alpha = 64.1791 | OR data |
| Serious adverse event → Death (SOC) | 0.2836 | 0.0448 | Dirichlet | alpha = 28.3582 | OR data |
| Serious adverse event → Treatment failure (unresolved) (SOC) | 0.0746 | 0.0261 | Dirichlet | alpha = 7.4627 | OR data |
| **Treatment completed (SOC)** |  |  |  |  |  |
| Treatment completed → Relapse (SOC) | 0.0014 | 0.0037 | Dirichlet | alpha = 0.1366 | OR data |
| Treatment completed → Long-term cure (after 12 months) (SOC) | 0.0829 | 0.0274 | Dirichlet | alpha = 8.2916 | OR data |
| Treatment completed → Death (SOC) | 0.0036 | 0.0060 | Dirichlet | alpha = 0.3643 | OR data |
| Remaining in Treatment completed state (SOC) | 0.9121 | 0.0282 | Dirichlet | alpha = 91.2075 | OR data |
| **Common transitions** |  |  |  |  |  |
| **Subsequent SL treatment transitions** |  |  |  |  |  |
| SL treatment → Treatment failure | 0.0110 | 0.0104 | Dirichlet | alpha = 1.1033 | OR data |
| SL treatment → Successful treatment completion | 0.0399 | 0.0195 | Dirichlet | alpha = 3.9878 | OR data |
| SL treatment → Death during treatment | 0.0046 | 0.0068 | Dirichlet | alpha = 0.4644 | OR data |
| Remaining in SL treatment (subsequent regimens) state | 0.9444 | 0.0228 | Dirichlet | alpha = 94.4444 | OR data |
| **Lost to follow-up transitions** |  |  |  |  |  |
| Lost to follow-up → Switch to SL treatment (subsequent regimens) | 0.0230 | 0.0149 | Dirichlet | alpha = 2.3 | IHME, 2019 [13] |
| Lost to follow-up → Death | 0.0686 | 0.0252 | Dirichlet | alpha = 6.86 | Quaife, 2020 [12] |
| Remaining in Lost to follow-up state | 0.9084 | 0.0287 | Dirichlet | alpha = 90.84 | OR data |
| **Treatment failure (unresolved disease)** |  |  |  |  |  |
| Failure → Death | 0.0686 | 0.0252 | Dirichlet | alpha = 6.86 | Quaife, 2020 [12] |
| Remaining in Failure state | 0.9314 | 0.0252 | Dirichlet | alpha = 93.14 | OR data |

^a^ Gamma distributions were parameterised assuming a coefficient of variation of 20% (shape = 25). ^b^ Dirichlet distributions were applied to sets of mutually exclusive transition probabilities constrained to sum to one. For transition sets modelled using Dirichlet distributions, individual rows present component-specific alpha values, while complete alpha vectors comprise all competing outcomes within the corresponding transition block. Alpha parameters were calculated as base-case probabilities × 100. Standard deviations represent the theoretical marginal SD of each Dirichlet component, calculated as √[p(1−p)/(α₀+1)], where α₀ is the sum of alpha parameters [15].
