# Supplementary material for: Cost-effectiveness of BPaL-based and 9-month modified all-oral short treatment regimens for rifampicin-resistant tuberculosis in Belarus
Source: PLOS Glob Public Health. 2026 Jul 23;6(7):e0005872. doi: 10.1371/journal.pgph.0005872 (PMC13395433; doi:10.1371/journal.pgph.0005872)
Supplement: S5 Table — (DOCX) [file pgph.0005872.s011.docx]

**S5 Table. Expected clinical outcomes over a 20-year horizon (per 1,132 patients).**

| **Outcome** | **BPaL(M/C)** | **mSTR** | **SOC** |
| --- | --- | --- | --- |
| Cured, % (95% CI) | 87.7 (85.7; 89.5) | 83.3 (81.0; 85.4) | 68.3 (65.5; 70.9) |
| Death, % (95% CI) | 12.3 (10.5; 14.3) | 16.7 (14.6; 19.0) | 31.7 (29.1; 34.5) |
| Treatment failure, % | 0.0 | 0.0 | 0.0 |
| Lost to follow-up, % | 0.0 | 0.0 | 0.0 |

Due to the 20-year model horizon, initial treatment failures and loss to follow-up cases either transition to successful treatment following re-treatment or result in death, resulting in negligible proportions for these categories at the end of the simulation.
