# Supplementary material for: Cost-effectiveness of BPaL-based and 9-month modified all-oral short treatment regimens for rifampicin-resistant tuberculosis in Belarus
Source: PLOS Glob Public Health. 2026 Jul 23;6(7):e0005872. doi: 10.1371/journal.pgph.0005872 (PMC13395433; doi:10.1371/journal.pgph.0005872)
Supplement: S6 Table — (DOCX) [file pgph.0005872.s012.docx]

**S6 Table. Univariate sensitivity analyses – impact on incremental costs, QALYs, and INMB.**

| Parameter | Parameter change | Incremental Cost, USD | Incremental QALYs | INMB | Incremental Cost, USD | Incremental QALYs | INMB | Incremental Cost, USD | Incremental QALYs | INMB |
| --- | --- | --- | --- | --- | --- | --- | --- | --- | --- | --- |
|  |  | mSTR vs SOC | | | BPaL(M/C) vs SOC | | | BPaL(M/C) vs mSTR | | |
| **Utilities** | | | | | | | | | | |
| Utility (adverse health states: SAE/LTFU/Unresolved) | −0.14 | −11,560.2 | 1.4598 | 20,468 | −12,602.7 | 1.9271 | 24,362 | −1,042.5 | 0.4673 | 3,894 |
|  | +0.14 | −11,560.2 | 1.4342 | 20,312 | −12,602.7 | 1.8984 | 24,186 | −1,042.5 | 0.4642 | 3,875 |
| Utility (favourable health states: on-treatment/treatment completed/cured) | −0.04 | −11,560.2 | 1.3597 | 19,857 | −12,602.7 | 1.8005 | 23,589 | −1,042.5 | 0.4408 | 3,732 |
|  | +0.04 | −11,560.2 | 1.5087 | 20,766 | −12,602.7 | 1.9962 | 24,784 | −1,042.5 | 0.4875 | 4,017 |
| **Costs** | | | | | | | | | | |
| Non-drug costs BPaL(M/C) treatment | −25% | — | — | — | −12,836.5 | 1.9127 | 24,508 | −1,276.3 | 0.4657 | 4,118 |
|  | +25% | — | — | — | −12,368.9 | 1.9127 | 24,040 | −808.7 | 0.4657 | 3,651 |
| Non-drug costs mSTR treatment | −25% | −11,892.0 | 1.4470 | 20,721 | — | — | — | −710.8 | 0.4657 | 3,553 |
|  | +25% | −11,228.6 | 1.4470 | 20,058 | — | — | — | −1,374.2 | 0.4657 | 4,216 |
| Non-drug costs SOC treatment | −25% | −9,858.1 | 1.4470 | 18,688 | −10,900.5 | 1.9127 | 22,572 | — | — | — |
|  | +25% | −13,262.4 | 1.4470 | 22,092 | −14,304.9 | 1.9127 | 25,976 | — | — | — |
| Costs SL subsequent regimen | −25% | −11,474.5 | 1.4470 | 20,304 | −12,462.2 | 1.9127 | 24,134 | −987.8 | 0.4657 | 3,830 |
|  | +25% | −11,646.0 | 1.4470 | 20,476 | −12,743.2 | 1.9127 | 24,415 | −1,097.2 | 0.4657 | 3,939 |
| **Costs and utilities** | | | | | | | | | | |
| Discount rate | 0% | −12,473.1 | 2.0194 | 24,796 | −13,628.8 | 2.6583 | 29,850 | −1,155.7 | 0.6389 | 5,054 |
|  | 6% | −10,753.6 | 1.0614 | 17,230 | −11,691.3 | 1.4096 | 20,293 | −937.6 | 0.3482 | 3,063 |
