# Supplementary material for: Cost-effectiveness of BPaL-based and 9-month modified all-oral short treatment regimens for rifampicin-resistant tuberculosis in Belarus
Source: PLOS Glob Public Health. 2026 Jul 23;6(7):e0005872. doi: 10.1371/journal.pgph.0005872 (PMC13395433; doi:10.1371/journal.pgph.0005872)
Supplement: S7 Table — (DOCX) [file pgph.0005872.s013.docx]

**S7 Table. Univariate sensitivity analyses – impact on incremental net monetary benefit (INMB).**

| **Sensitivity analyses** | **Parameter change** | **INMB** | | |
| --- | --- | --- | --- | --- |
|  |  | **mSTR vs SOC** | **BPaL(M/C) vs SOC** | **BPaL(M/C) vs mSTR** |
| Regimen cost (≥18-month regimen) | +25% | 25,255.5 | 29,139.9 | — |
| Regimen cost (≥18-month regimen) | -25% | 15,524.2 | 19,408.7 | — |
| Regimen cost (mSTR) | +25% | 18,310.2 | — | 5,964.1 |
| Regimen cost (mSTR) | -25% | 22,469.5 | — | 1,804.8 |
| Regimen cost (BPaL(M/C)) | +25% | — | 22,403.7 | 2,013.9 |
| Regimen cost (BPaL(M/C)) | -25% | — | 26,144.9 | 5,755.1 |
| SAE on treatment (≥18-month regimen) | +25% | 21,247.0 | 25,131.5 | — |
| SAE on treatment (≥18-month regimen) | -25% | 19,487.0 | 23,371.5 | — |
| SAE on treatment (mSTR) | +25% | 19,690.2 | — | 4,584.1 |
| SAE on treatment (mSTR) | -25% | 21,109.4 | — | 3,165.0 |
| SAE on treatment (BPaL(M/C)) | +25% | — | 23,904.0 | 3,514.2 |
| SAE on treatment (BPaL(M/C)) | -25% | — | 24,649.6 | 4,259.8 |
| Relapse during post-treatment follow-up (≥18-month regimen) | +25% | 20,473.5 | 24,358.0 | — |
| Relapse during post-treatment follow-up (≥18-month regimen) | -25% | 20,311.1 | 24,195.6 | — |
| Relapse during post-treatment follow-up (mSTR) | +25% | 20,350.3 | — | 3,924.1 |
| Relapse during post-treatment follow-up (mSTR) | -25% | 20,428.5 | — | 3,845.8 |
| Relapse during post-treatment follow-up (BPaL(M/C)) | +25% | — | 24,190.2 | 3,800.4 |
| Relapse during post-treatment follow-up (BPaL(M/C)) | -25% | — | 24,354.7 | 3,964.9 |
| Second-line treatment cost (subsequent regimen) | +25% | 20,475.6 | 24,414.8 | 3,939.2 |
| Second-line treatment cost (subsequent regimen) | -25% | 20,304.1 | 24,133.8 | 3,829.8 |
| Mortality in LTFU | +25% | 20,500.7 | 24,368.6 | 3,867.9 |
| Mortality in LTFU | -25% | 20,232.1 | 24,140.3 | 3,908.2 |
| Utility (adverse health states: SAE/LTFU/Unresolved) | +0.14 | 20,311.6 | 24,186.5 | 3,875.0 |
| Utility (adverse health states: SAE/LTFU/Unresolved) | -0.14 | 20,468.1 | 24,362.1 | 3,894.0 |
| Utility (treatment completed) | +0.04 | 20,429.5 | 24,326.5 | 3,896.9 |
| Utility (treatment completed) | -0.04 | 20,350.1 | 24,222.2 | 3,872.0 |
