# Supplementary material for: Cost-effectiveness of BPaL-based and 9-month modified all-oral short treatment regimens for rifampicin-resistant tuberculosis in Belarus
Source: PLOS Glob Public Health. 2026 Jul 23;6(7):e0005872. doi: 10.1371/journal.pgph.0005872 (PMC13395433; doi:10.1371/journal.pgph.0005872)
Supplement: Attachment — Submitted filename: Review PGPH-D-26-00136.pdf [file pgph.0005872.s014.pdf]

Review

Manuscript ID: PGPH-D-26-00136
Paper Title: Cost-effectiveness of BPaL-based and 9-month modified all-oral short treatment regimens for rifampicin-resistant tuberculosis

The author uses a Markov cohort model to evaluate the cost-effectiveness of shorter treatment regimens for rifampicin-resistant tuberculosis in Belarus. Overall, the study is conducted well. My main comments concern how the CEA results are presented.

1. In the abstract and a few other places, the CEA results are summarized based on the percentage of simulations where BPaL(M/C) and mSTR were cost-effective. This is mainly rooted in the use of cost-effectiveness acceptability curves (CEAC). I understand that CEAC is a popular method for presenting the results of a probabilistic sensitivity analysis. However, mounting evidence suggests that CEAC may be misleading. For example, refer to https://pubmed.ncbi.nlm.nih.gov/17409361/ and https://pubmed.ncbi.nlm.nih.gov/39056310/. If the author wishes to still present CEAC, that is ok, but I would encourage the author to put less emphasis on what CEAC shows (and to move CEAC to the supplement).
2. A more appropriate way to present the results of CEA is to use:
    a. Cost-effectiveness plane, where the origin represents the SOC,
    b. Cost-effectiveness table, where dominated or cost-saving strategies are clearly marked, and the ICER for the non-dominated strategies is presented,
    c. Incremental NMB lines.

    The author is already using some of these tools, but a few modifications might be needed:
    1. Decision-making should be based on the *expected* value of outcomes (and not the median). See https://pubmed.ncbi.nlm.nih.gov/10537899/ for example. Hence, triangles in Fig. 4 should represent the expected values (the green triangle should be at the origin).
    2. Table 2 resembles a CE table but is not accurate. First, the dominated strategies should be marked, and the ICER should be calculated only for non-dominated strategies. The CE table should be presented for both the main and conservative scenarios.
    3. Fig. 5 could be misleading since the alternatives are compared pairwise. It should be modified to reflect the incremental NMB for each alternative relative to the SOC/baseline. It should also show the expected values rather than the medians. See https://pubmed.ncbi.nlm.nih.gov/39056310/ for example.
3. I could not find the list of parameters along with the probability distributions used to model uncertainties in their estimates. If this is not presented, please include a table or tables listing the parameters, the assumed probability distributions, and the sources that inform these distributions.
4. The results are mainly focused on the CEA outcomes. It would also be helpful to present some intermediate outcomes, such as the expected number of people successfully treated under each regimen.

Other comments:

1. It would be helpful to add a Markov diagram to more clearly present the assumed health states and the transition between them.
2. It is stated in lines 60–61 that “Patients were considered cured if they remained in the treatment completion state for 10 consecutive months without relapse …”. Given that the author is using a cohort Markov model, I am not sure how this assumption is modeled.
3. It might be helpful to be explicit in the paper title that the study is focused on Belarus.
