# Supplementary material for: Cost-effectiveness of BPaL-based and 9-month modified all-oral short treatment regimens for rifampicin-resistant tuberculosis in Belarus
Source: PLOS Glob Public Health. 2026 Jul 23;6(7):e0005872. doi: 10.1371/journal.pgph.0005872 (PMC13395433; doi:10.1371/journal.pgph.0005872)
Supplement: Attachment — Submitted filename: Review PGPH-D-26-00136R1.pdf [file pgph.0005872.s016.pdf]

Review

Manuscript ID: PGPH-D-26-00136R1
Paper Title: Cost-effectiveness of BPaL-based and 9-month modified all-oral short treatment regimens for rifampicin-resistant tuberculosis in Belarus

I appreciate the author's willingness to address my comments. The manuscript has improved, and the inclusion of additional information has helped, but also revealed some additional concerns.

1. It seems that CEA results are generated using the deterministic analysis (not including the uncertainties in parameters). Please note that CEA results (cost and QALYs, incremental cost and QALYs, and ICER, if applicable) should be based on the probabilistic sensitivity analysis (PSA). Please refer to the book "Decision Modelling for Health Economic Evaluation" by Briggs etc. In other words, PSA results are the main results to highlight and discuss.
2. The inclusion of the model diagram has been helpful, and I would recommend moving it into the main text. However, it would be helpful to define each health state more clearly:
    a. What is the distinction between "BPaL(M/C) or mSTR or SOC" state and the "On Treatment" State? Why is the distinction important?
    b. "Treatment failure" seems to be an absorbing state. What happens to patients who experience treatment failure in reality?
3. It is important to display the trajectories of this model (e.g., number of individuals in each health state over time) to make sure the model is behaving as expected. This is particularly important for PSA to make sure model trajectories remain reasonable when parameter values are drawn from distributions.
4. It is not clear whether background (non-TB-related) mortality is incorporated. Without that, the model might overestimate QALYs.
5. The cohort is simulated for 20 years, but given that this is a closed cohort, it might need to be simulated for a longer time to properly accumulate the cost and QALYs of this cohort. Displaying the model trajectories could help inform the simulation duration.
6. The list of model parameters in Table 1 does not clearly correspond to the Markov diagram, which creates confusion. The reader should be able to easily find the transition probabilities in the Markov diagram using the provided parameter table.
7. Most importantly, it is confusing to see two separate parameter tables, one in the main text and one in the supplement. When parameters are uncertain, PSA results are treated as the main results, so I recommend that the author present only the parameter table used in PSA.
8. Lines 81–83, please make sure these values are consistent with what is presented in the parameter table.
9. It is not clear how Table S2 is used in the analysis.

10. Table S3 provides the parameters of the probability distributions, which is helpful. However, providing the mean and the standard deviation of these distributions is more helpful and easier for the reader to evaluate whether the assumed distributions are justified. For example, it is not clear how the assumption that shape = 25 and scale = mean/25 impacts the mean and the st dev of the distribution.
11. Lines 115-117, this approach to sampling from transition probabilities is not correct. Since the sum of probabilities should add to 1, the uncertainty in transition probabilities must be modelled using Dirichlet distributions. Please refer to the book "Decision Modelling for Health Economic Evaluation" by Briggs etc.
12. The treatment duration for BPaL(M/C) provided in Table 1 is different from the standard duration of BPaL(M/C). Is this based on what is observed in Belarus?
13. Line 170, Figure S2 doesn't show the CE frontier. Both Figure S2 and S3 appear to be based on deterministic analysis, which may be misleading when parameter values are uncertain.
