# Supplementary material for: Cost-effectiveness of BPaL-based and 9-month modified all-oral short treatment regimens for rifampicin-resistant tuberculosis in Belarus
Source: PLOS Glob Public Health. 2026 Jul 23;6(7):e0005872. doi: 10.1371/journal.pgph.0005872 (PMC13395433; doi:10.1371/journal.pgph.0005872)
Supplement: Attachment — Submitted filename: Response to Reviewers.docx [file pgph.0005872.s017.docx]

**Manuscript ID:** PGPH-D-26-00136

**Title:** Cost-effectiveness of BPaL-based and 9-month modified all-oral short treatment regimens for rifampicin-resistant tuberculosis in Belarus

We thank the Editor and Reviewers for their thoughtful and constructive comments. We have carefully revised the manuscript to address all comments raised. We believe these revisions have strengthened the clarity, methodological transparency, and presentation of the cost-effectiveness analysis.

All changes are highlighted in the revised manuscript. A point-by-point response is provided below.

We thank the Reviewer for this important comment regarding the interpretation of cost-effectiveness acceptability curves (CEACs).

**Comment 1 — CEAC emphasis**

Response:

1. In response, we have revised the manuscript to reduce the emphasis on CEAC-based interpretation. Specifically, we have:

- shifted the primary interpretation of probabilistic results towards expected values of costs and QALYs and incremental net monetary benefit (INMB),

- clarified the role of CEAC as a complementary descriptive tool rather than a basis for decision-making, and

- moved the CEAC figure to the Supplementary Materials.

These changes align the presentation more closely with recommended approaches to decision-making under uncertainty. These revisions ensure that decision-making is based on expected values rather than distributional summaries.

**Comment 2.1 — Expected values vs medians**

**Comment 2.2 — Table 2 (CE table)**

**Comment 2.3 — Figure 5 (INMB)**

Response:

2. We thank the Reviewer for highlighting the importance of expected values in economic evaluation.

a. We have revised the analysis and presentation to report mean (expected) costs, QALYs, and incremental outcomes as the primary basis for decision-making.

Figures and tables have been updated accordingly. In particular, the cost-effectiveness plane (now Figure 3) now presents expected values, with the origin corresponding to the standard of care (SOC).

b. We have revised the presentation of cost-effectiveness results by providing separate tables for both the base-case (Table 2) and the conservative scenario (now Table 3), ensuring alignment with standard reporting conventions. Strategies are now evaluated using standard dominance rules.

- Strategies are now ordered by total costs.
- Regimens that were both more effective and less costly (base-case) are explicitly labeled as "Dominant".
- In the conservative scenario, where regimens were less costly but slightly less effective, they are labeled as "Cost-saving".
- Numerical ICERs were omitted for these strategies to avoid methodological inconsistency associated with negative incremental values; instead, the Incremental Net Monetary Benefit (INMB) is presented to provide a robust integrated measure.

We thank the Reviewer for this point. In the revised manuscript, we have explicitly identified the standard of care (SOC) as a dominated strategy in the Results section for the base-case analysis.

c. We thank the Reviewer for this suggestion.

Figure 4 has been revised to present incremental net monetary benefit (INMB) for each strategy relative to the standard of care, rather than pairwise comparisons. The figure now reflects expected values across willingness-to-pay thresholds, consistent with decision-analytic recommendations.

This revision improves interpretability and alignment with the decision context.

**Comment 3 — Parameter distributions**

Response:

3. We agree that transparent reporting of parameter uncertainty is essential.

We have added a new Supplementary Table (S3 Table), which provides a comprehensive list of model parameters, including point estimates, probability distributions, parameterisation, and data sources.

This addition improves the transparency and reproducibility of the analysis.

**Comment 4 — Intermediate outcomes**

Response:

4. We thank the Reviewer for this suggestion.

We have expanded the Results section and added a new Supplementary Table (S5 Table) to include intermediate clinical outcomes, providing the expected proportion of patients successfully treated under each regimen. These results provide additional clinical context to complement the economic findings.

**Other comment 1 — Markov diagram**

Response:

We have added a Markov model diagram to the Supplementary Materials (S1 Figure) to more clearly illustrate health states and transitions.

**Other comment 2 — Cure definition**

Response:

We have clarified the definition and implementation of “cure” within the cohort Markov framework in the Methods section.

Specifically, we explain that the 12-month follow-up period is operationalised as a transitional state with a monthly probability of 1/12 (0.0833) to ensure the correct mean residence time.

We have also revised the Abstract and Results to align with these changes.

**Other comment 3 — Title (Belarus)**

Response:

We agree that specifying the study setting improves clarity.

As suggested, we have updated the title of the manuscript to specifically include 'Belarus' to better reflect the geographical context of the study.

We thank Reviewer #2 for the positive and supportive evaluation of our manuscript.

We hope that the revisions adequately address the Reviewer’s comments and improve the manuscript. We would be happy to provide any further clarification if needed.
