# Supplementary material for: Cost-effectiveness of BPaL-based and 9-month modified all-oral short treatment regimens for rifampicin-resistant tuberculosis in Belarus
Source: PLOS Glob Public Health. 2026 Jul 23;6(7):e0005872. doi: 10.1371/journal.pgph.0005872 (PMC13395433; doi:10.1371/journal.pgph.0005872)
Supplement: Attachment — Submitted filename: RESPONSE TO REVIEWER.docx [file pgph.0005872.s018.docx]

**RESPONSE TO REVIEWER**

**Manuscript ID:** PGPH-D-26-00136

**Title:** Cost-effectiveness of BPaL-based and 9-month modified all-oral short treatment regimens for rifampicin-resistant tuberculosis in Belarus

We thank the Editor and Reviewers for their thoughtful and constructive comments. We have carefully revised the manuscript to address all comments raised. We believe these revisions have strengthened the clarity, methodological transparency, and presentation of the cost-effectiveness analysis.

All changes are highlighted in the revised manuscript. A point-by-point response is provided below.

**Reviewer Comment 1**

It seems that CEA results are generated using the deterministic analysis... PSA results are the main results to highlight and discuss.

**Response:**

We thank the reviewer for this important methodological comment. We agree that probabilistic sensitivity analysis (PSA) provides the primary basis for decision-making under parameter uncertainty. Accordingly, we have revised the manuscript to give greater prominence to PSA findings in the Results, Abstract, and Discussion sections. Base-case deterministic results are now presented as reference estimates, while PSA results are emphasised as the principal cost-effectiveness findings.

**Reviewer Comment 2a**

What is the distinction between “BPaL(M/C) or mSTR or SOC” state and the “On Treatment” state?

**Response:**

We thank the reviewer for highlighting the need for clearer state definitions. We have revised the figure caption and Methods section to clarify this distinction. The initial strategy state (“BPaL(M/C), mSTR, or SOC”) denotes the treatment regimen assigned at model entry and determines regimen-specific duration, costs, effectiveness, and safety parameters. The “On treatment” state represents patients actively receiving therapy following treatment initiation, including continuation or resumption of treatment after loss to follow-up or serious adverse events.

**Reviewer Comment 2b**

“Treatment failure” seems to be an absorbing state. What happens to patients who experience treatment failure in reality?

**Response:**

We thank the reviewer for this important observation. In the model, the “treatment failure” state represents patients with persistent disease after exhaustion of available effective treatment options. In current practice, such patients may receive supportive or palliative care, including clinical monitoring and symptomatic management, rather than further curative therapy. This was therefore represented as a non-curative absorbing state associated with ongoing mortality risk.

**Reviewer Comment 3**

It is important to display the trajectories of this model...

**Response:**

We agree and have added a new figure presenting modelled cohort trajectories across health states over the 20-year horizon for each regimen. This figure shows that most transitions occurred within the first years after treatment initiation, with outcomes stabilising thereafter, supporting the face validity of model behaviour and suggesting that the selected time horizon was sufficient.

**Reviewer Comment 4**

It is not clear whether background mortality is incorporated.

**Response:**

We thank the reviewer for this important comment. In response, we revised the model to explicitly incorporate mortality during the 12-month post-treatment follow-up period before transition to the cured state. Patients completing treatment therefore remained at risk of both relapse and death during follow-up and were classified as cured only if they remained alive and relapse-free throughout this period. Patients entering the cured state were assumed to remain free of further TB-related events for the remainder of the model horizon. Background non-TB mortality after cure was not explicitly modelled; however, because this assumption was applied equally across all strategies, it is unlikely to materially affect the comparative cost-effectiveness results.

**Reviewer Comment 5**

The cohort is simulated for 20 years...

**Response:**

We thank the reviewer for this helpful suggestion. The newly added cohort trajectory figure shows that nearly all clinically relevant transitions occurred during the early years of simulation, with outcomes stabilising well before the end of the 20-year horizon. These findings indicate that extending the time horizon further would be unlikely to materially affect the comparative cost-effectiveness results. We therefore consider the selected horizon sufficient to capture relevant long-term costs and health outcomes.

**Reviewer Comment 6**

The list of model parameters in Table 1 does not clearly correspond to the Markov diagram.

**Response:**

We thank the reviewer for this helpful comment. In response, we substantially revised Table 1 to improve consistency with the Markov model structure and facilitate interpretation of model inputs. The table is now organised into clearly defined sections covering costs, health-state utility weights, treatment outcomes, SAE outcomes, and follow-up outcomes used to parameterise model transitions. Outcome parameters are presented using descriptive labels that correspond directly to pathways shown in the Markov diagram, allowing readers to more easily identify how model transitions were parameterised.

We additionally reviewed and harmonised regimen-specific operational research inputs to ensure internal consistency across treatment outcomes, SAE outcomes, and post-treatment follow-up outcomes.

**Reviewer Comment 7**

It is confusing to see two separate parameter tables...

**Response:**

We thank the reviewer for this helpful suggestion. We have revised the presentation of the tables to better distinguish their respective purposes. Table 1 now summarises the key clinical and economic model inputs used in the base-case analysis, including treatment outcomes, health-state utility weights, and costs. The supplementary PSA table provides the probability distributions, parameterisation, and uncertainty ranges assigned to these inputs for probabilistic sensitivity analysis. This separation improves readability while preserving full transparency regarding model inputs and uncertainty assumptions.

**Reviewer Comment 8**

Lines 81–83, please make sure these values are consistent...

**Response:**

We thank the reviewer for noting this point. We reviewed the corresponding parameters and confirmed that the values reported in the manuscript text are consistent with those presented in the revised parameter tables.

**Reviewer Comment 9**

It is not clear how Table S2 is used in the analysis.

**Response:**

We appreciate this comment and have clarified the purpose of Table S2 in the supplementary materials. Table S2 presents the individual regimens included in the standard-of-care (SOC) comparator, together with their observed frequencies and associated costs. These data were used to calculate the weighted average drug acquisition cost of the SOC comparator incorporated into the economic model.

**Reviewer Comment 10**

Providing the mean and standard deviation would be more helpful...

**Response:**

We thank the reviewer for this useful recommendation. We have revised the supplementary PSA parameter table to additionally report base-case values together with standard deviations, alongside the distribution type and parameterisation. This should make the implied uncertainty assumptions easier to interpret.

**Reviewer Comment 11**

This approach to sampling transition probabilities is not correct... Dirichlet distributions should be used.

**Response:**

We thank the reviewer for this important methodological observation. We agree that mutually exclusive transition probabilities constrained to sum to one are more appropriately represented using Dirichlet distributions. We have therefore revised the probabilistic sensitivity analysis accordingly and implemented Dirichlet distributions for all relevant sets of competing transition probabilities. The Methods section and supplementary PSA parameter table have been updated to reflect this change, including the corresponding parameterisation and uncertainty assumptions.

**Reviewer Comment 12**

The treatment duration for BPaL(M/C)... different from standard duration.

**Response:**

We thank the reviewer for this question. Yes, the 24-week duration reflects the regimen as implemented under Belarus national operational research conditions (SMARRTT), informed by TB-PRACTECAL, rather than the 26-week duration recommended by the World Health Organization. The economic evaluation was therefore based on the observed implementation duration in Belarus. We have clarified this in the Methods section.

**Reviewer Comment 13**

Figure S2 doesn’t show the CE frontier...

**Response:**

We thank the reviewer for this helpful comment. We agree that the previous terminology could be misleading. The figure title and text have been revised accordingly. The figure is now labelled as a cost-effectiveness plane showing expected costs and QALYs, rather than a cost-effectiveness frontier.
